# Immunometabolism: signaling pathways, homeostasis, and therapeutic targets

**DOI:** 10.1002/mco2.789

**Published:** 2024-11-03

**Authors:** Rongrong Xu, Xiaobo He, Jia Xu, Ganjun Yu, Yanfeng Wu

**Affiliations:** ^1^ National Key Laboratory of Immunity and Inflammation & Institute of Immunology College of Basic Medical Sciences Naval Medical University Shanghai China; ^2^ School of Life Sciences Fudan University Shanghai China

**Keywords:** immunometabolism, immune cells, immunometabolic enzymes, homeostasis, checkpoint

## Abstract

Immunometabolism plays a central role in sustaining immune system functionality and preserving physiological homeostasis within the organism. During the differentiation and activation, immune cells undergo metabolic reprogramming mediated by complex signaling pathways. Immune cells maintain homeostasis and are influenced by metabolic microenvironmental cues. A series of immunometabolic enzymes modulate immune cell function by metabolizing nutrients and accumulating metabolic products. These enzymes reverse immune cells’ differentiation, disrupt intracellular signaling pathways, and regulate immune responses, thereby influencing disease progression. The huge population of immune metabolic enzymes, the ubiquity, and the complexity of metabolic regulation have kept the immune metabolic mechanisms related to many diseases from being discovered, and what has been revealed so far is only the tip of the iceberg. This review comprehensively summarized the immune metabolic enzymes’ role in multiple immune cells such as T cells, macrophages, natural killer cells, and dendritic cells. By classifying and dissecting the immunometabolism mechanisms and the implications in diseases, summarizing and analyzing advancements in research and clinical applications of the inhibitors targeting these enzymes, this review is intended to provide a new perspective concerning immune metabolic enzymes for understanding the immune system, and offer novel insight into future therapeutic interventions.

## Graphical abstract

Metabolic enzymes can directly or indirectly impact the differentiation, activation, and proliferation of immune cells through the metabolites generated by their enzymatic activities or nonenzymatic signal interference, thereby modulating the function of immune cells. Currently, a variety of inhibitors or antibodies targeting metabolic enzymes have been developed for the treatment of multiple myeloma, melanoma, glioma, and other diseases

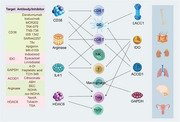

## 1 INTRODUCTION

The immune system acts as the sentinel of the body, interfacing with other physiological systems, such as the nervous and endocrine systems, through intricate interactions to form a harmonized biodefense network.^1^, ^2^ Within this network, immune cells, as the core executors of the immune system, ensure the balance and stability of the body's immune responses through a series of complex and precise regulatory mechanisms. This coordination is vital for maintaining health, the defense against pathogens, and the overall surveillance against threats to homeostasis.

The complexity of the immune system is not only evident in its cellular diversity and intricate signaling networks but also reflected in its close interconnection with metabolic processes. The initiation and sustenance of immune responses fundamentally rely on metabolic processes, which necessitate ample energy provision and biosynthetic precursors to support the proliferation, differentiation, and effector functions of immune cells.^3^, ^4^, ^5^ Consequently, the metabolic milieu of the organism exerts a decisive influence on the activity and functional expression of immune cells.^6^ Metabolic pathways provide not only the essential energy and building blocks but also engage in the transmission and processing of signals within and outside immune cells through the agency of metabolic intermediates acting as signaling molecules.^7^ Immune cells maintain metabolic balance through homeostatic regulation, yet they are also subject to the influence of the microenvironment on cellular metabolism. Thus, immune cells are exceedingly sensitive to nutritional status and metabolic fluctuations, which together shape the quality and efficiency of immune reactions. Any imbalance in the metabolic state can significantly affect the functionality of immune cells, thereby impacting the overall efficacy of the immune response. The intricate interplay between cellular metabolism and immune function has emerged as a critical area of investigation in recent years.

In the domain of treating malignant disorders such as malignant tumors, researchers have increasingly and profoundly acknowledged that the loss or malfunction of immune cells constitutes a crucial factor influencing the treatment outcome.^8^ This malfunction of immune cells is frequently associated with metabolic disturbances, namely, immune cells are unable to exert their due antitumor activity due to the disorder of metabolic pathways or inadequate nutrient supply.^6^, ^9^ The metabolic impact might arise from the competitive pressure of the tumor microenvironment, metabolic reprogramming under chronic inflammatory circumstances, or the appropriation of nutrients by the tumor itself.^10^, ^11^, ^12^ Hence, comprehending and resolving the metabolic disorders of immune cells has emerged as an important strategy to enhance the efficacy of immunotherapy.

**FIGURE 1:**
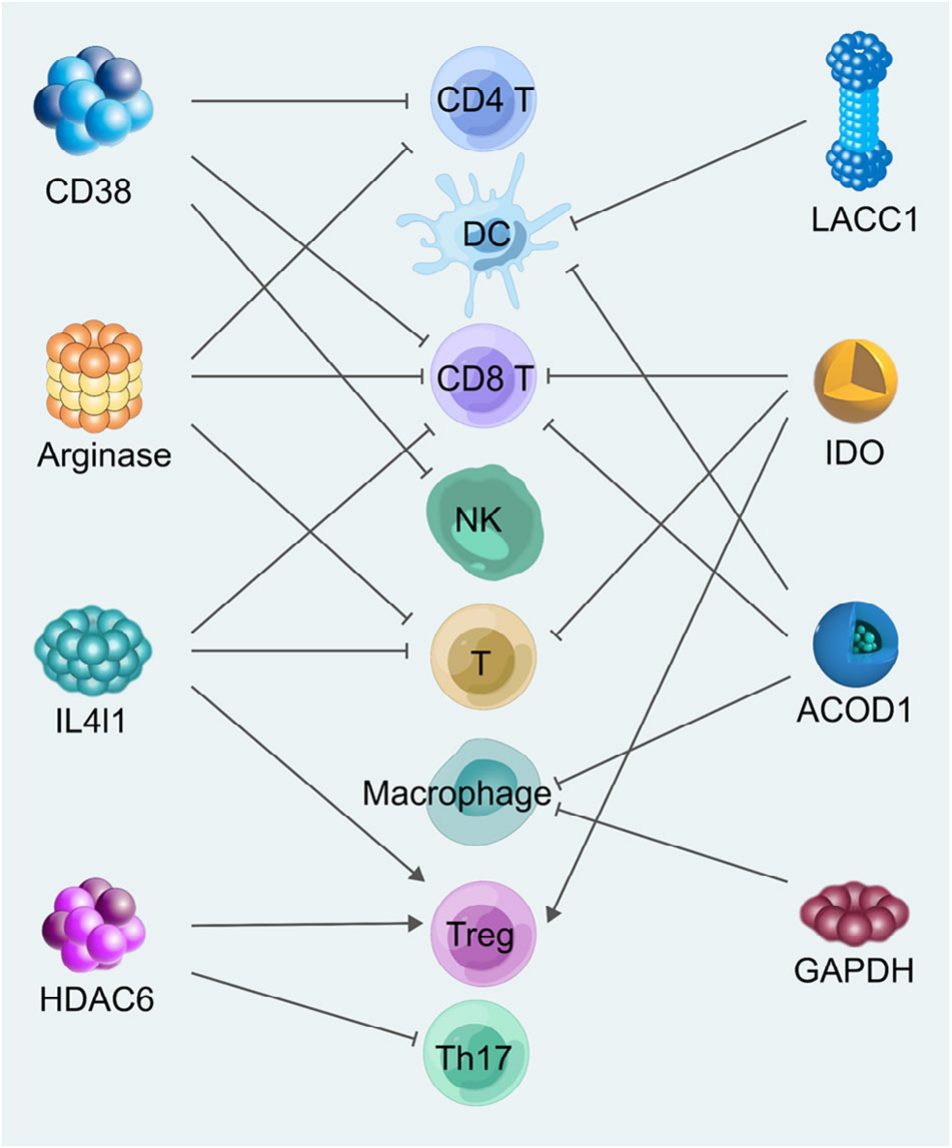
Metabolic enzymes modulate various immune cells. A variety of metabolic enzymes regulate the activity and proliferation of immune cells, impact cell function, and mediate immunosuppression through enzyme activity metabolites or nonenzymatic signal interference.

In this comprehensive review, we meticulously delineate the intricate signaling pathways that govern the development of immune cells, highlighting their pivotal role in sculpting the metabolic landscape of these cells. Furthermore, we conduct an exhaustive examination of the enzymatic profiles that significantly influence the metabolic machinery of immune cells, elucidating their crucial contributions to the orchestration of immune responses. Specifically, we discuss the roles of some conspicuous enzymes such as CD38, arginase, histone deacetylase 6 (HDAC6), interleukin‐4 induced 1 (IL4I1), indoleamine 2,3‐dioxygenase 1/2 (IDO1/2), tryptophan 2,3‐dioxygenase (TDO), laccase domain‐containing 1 (LACC1), glyceraldehyde‐3‐phosphate dehydrogenase (GAPDH), and aconitate decarboxylase 1 (ACOD1). We have analyzed and organized information on the structure, distribution, metabolic substrates, and products of these enzymes, as well as their multifaceted effects on immune cells (Figure 1). Ultimately, the review conducts an in‐depth exploration of targeted therapies for immunometabolic enzymes, encompassing inhibitors, antibodies, and chimeric antigen receptor (CAR) therapies. By concentrating on the intricate interplay between immune cell metabolism and enzymatic targets, the review underscores the promise of these strategies in enhancing therapeutic efficacy and offers a roadmap for future research and clinical applications.

## 2 SIGNALING PATHWAYS OF REGULATING IMMUNOMETABOLISM IN IMMUNE CELLS DIFFERENTIATION AND FUNCTION

Immune cells stand as the fundamental pillars of the immune system, fulfilling essential roles in immune defense, surveillance, and regulation within the body. The differentiation and maturation of immune cells are governed by an elaborately regulated, dynamic process. Signaling pathways and cytokines involved in the differentiation, development, and activation of immune cells work in concert to form an intricate regulatory network. This network ensures the precision and efficacy of the immune system. Throughout this process, metabolic changes often accompany cellular transformations, reflecting the complex interplay between cellular function and metabolic homeostasis.

### 2.1 Immunometabolism modulation in T cells differentiation and function

T cells are derived from the bone marrow's pluripotent hematopoietic stem cells (HSCs) and their lymphoid progeny. Upon egress from the bone marrow, these lymphoid progenitor cells migrate to the thymus, where they undergo a critical phase of T cell development characterized by the rearrangement of T cell receptor (TCR) genes. Thymocytes that express both the TCR β chain and the CD4 and CD8 coreceptors are termed double‐positive (DP) cells.^13^ Before the β selection, thymocytes remain relatively quiescent, relying on highly efficient and energy‐saving oxidative metabolism. Following β selection, pre‐TCR signaling, Notch, and IL‐7 signals activate the PI3K signaling pathway. Subsequently, Notch signaling and the PI3K–Akt cascade further activate glucose transporter type 1 (Glut1), leading to glycolysis.^14^, ^15^, ^16^ In cells that have undergone β‐selection, the rearrangement of the TCRα gene segments is initiated, leading to the assembly of αβTCR heterodimers on the cell surface. This αβTCR complex is crucial for the provision of survival signals and for guiding the subsequent maturation and differentiation of the thymocytes into functional T cells.^13^ The protein kinase mammalian target of rapamycin (mTOR) primarily functions in regulating cellular growth and metabolism through two complexes, mTOR complex 1 (mTORC1) and mTORC2.^15^ The PI3K–Akt pathway activates the tuberous sclerosis complex, which activates Rheb within the lysosomes, ultimately leading to the activation of mTORC1.^17^ mTORC1 is particularly involved in the development of αβ T cells in the thymus. It integrates signals from the TCR and Notch, leading to the induction of the cellular myelocytomatosis oncogene (c‐Myc), a cytokine that facilitates lipid synthesis and ROS production. Furthermore, mTORC1 activates hypoxia‐inducible factor 1‐alpha (HIF1α), thereby increasing glycolytic metabolism and the pentose phosphate pathway (PPP).^15^

In the thymic environment, thymocytes that fail to express TCR are subjected to an intrinsic apoptotic mechanism. This process, known as “death‐by‐neglect,” is governed by the BCL‐2 family of proteins, which regulate cell survival and death. Thymocytes that do not successfully undergo TCR gene rearrangement and surface expression are consequently targeted for elimination, ensuring the maintenance of a functional and self‐tolerant T cell repertoire.^18^ Subsequently, the cells undergo a process of positive and negative selection based on their ability to react with the major histocompatibility complex (MHC) and peptide presented in the thymus with low affinity. During positive selection, cells that can bind to MHC class I molecules retain the expression of CD8, while the expression of CD4 diminishes; conversely, cells that can bind to MHC class II molecules maintain the expression of CD4 and lose CD8 expression. This selective process results in differentiated CD8^+^ and CD4^+^ single‐positive (SP) cells, which are pivotal for the adaptive immune response.^13^, ^19^, ^20^ During this process, regulatory factors c‐Myc and HIF1α are downregulated, leading to a sharp decrease in glucose metabolism within cells and a return to mitochondrial oxidative metabolism, which promotes the substantial generation of ATP.^15^

Beyond the TCR signaling pathway, the interaction between CD28 on the surface of thymocytes and its ligand B7 can significantly amplify TCR signals.^21^ This engagement of the CD28 costimulatory molecule with B7 family members on antigen‐presenting cells (APCs) provides a crucial second signal that enhances the survival and proliferation of T cells. This costimulation is essential for fully activating T cells, promoting their transition from a quiescent state to one of clonal expansion and differentiation, thereby bolstering the immune response to antigenic challenges. The CD28–B7 interaction is a key component of the intricate network of signals that drive T cell development and function within the thymic environment.^22^ CD28 costimulation also mediates the upregulation and facilitates the cellular surface transport of the glucose transporter Glut1 through Akt‐dependent and Akt‐independent pathways, enhancing glucose uptake following TCR stimulation1. This metabolic reprogramming is essential for the activation of T cells and the development of memory‐phenotype T cells.^23^ Glutamine, an indispensable amino acid for T cell activation, does not merely increase intake during T cell activation. Instead, it is through CD28 costimulation that the expression of glutamine transporters is enhanced, along with the activity of enzymes required to utilize glutamine as a substrate in the Krebs cycle within T cells. This intricate modulation ensures that the demand for glutamine is met during T cell activation.^24^, ^25^ Thymocyte differentiation and development, influenced by various signaling pathways, are accompanied by changes in cellular metabolic profiles. This metabolic shift allows these cells to meet the heightened energy requirements for their activation, proliferation, and differentiation into mature T cells, underscoring the importance of metabolic regulation in immune cell development and function.

After activation, CD4 T cells differentiate into multiple effector T cell subtypes, including regulatory T (Treg) cells, Th1, Th2, Th17, and so on. Once pathogens invade the body, the combination of TCR and the peptide–MHC II complex presented by APCs triggers the differentiation of Th1 cells. The IL‐12 and interferon‐γ (IFN‐γ) produced by APCs facilitate the further differentiation of T cells and activate signal transducer and activator of transcription 4 (STAT4) and STAT1. Under the combined effect of the transcription factor T‐bet induced by TCR stimulation, Th1 cells are prompted to produce IFN‐γ. The nuclear factor‐κB (NF‐κB) members c‐Rel and RelB also exert significant roles in the differentiation, development, and function of Th1. RelA is recruited to the IFN‐γ enhancer region in a T‐bet‐dependent manner and cooperates synergistically with STAT4 to enhance IFN‐γ expression. c‐Rel may act downstream of T‐bet, while RelB influences the expression of T‐bet and IFN‐γ via STAT4.^26^ Mannose metabolism is essential for the expression of IFN‐γ in Th1 cells.^27^ An intriguing finding suggests that the differentiation and effector functions of Th1 cells are mutually exclusive in terms of metabolic pathways. Only when the substrates produced by the initial proliferative and epigenetic reprogramming metabolic pathways required for TH1 cell activation are consumed by a different metabolic pathway, that is, after cell differentiation is suppressed, can the terminal effector functions of Th1 cells be exerted.^28^

Upon stimulation by IL‐4, CD4 T cells activate the transcription factor STAT6, which sets in motion the process of Th2 differentiation. This activation triggers a cascade of events, including the activation of the nuclear factor of activated T cells, activator protein‐1, and NF‐κB, as well as the induction of the pivotal transcription factor GATA3. GATA3 is responsible for the regulation of c‐Maf expression. STAT6, GATA3, and c‐Maf work in concert to reinforce the Th2 phenotype, ensuring the expression of signature cytokines and other genes characteristic of Th2 cells. Additionally, the NF‐κB family member p50 is a crucial component in the Th2 development pathway, playing an indispensable role in the differentiation and function of these cells. Th2 cells are critical for the immune response to parasites and allergens.^26^, ^29^ The differentiation of Th2 cells entails a metabolic adaptation, transitioning from energy acquisition through the tricarboxylic acid (TCA) cycle in CD4 T cells to a metabolic mode that relies heavily on fatty acid oxidation (FAO) for substantial energy production. mTORC1 facilitates the differentiation of Th2 cells. By activating the SREBP transcription factor, mTORC1 promotes the lipid synthesis pathway, meeting the lipid metabolic demands during Th2 cell activation. Among effector helper T cells, Th2 cells exhibit the highest expression of Glut1 and the most robust glycolytic effect. Peroxisome proliferator‐activated receptor gamma (PPAR‐γ) links Th2 cell function and cellular metabolism by regulating fatty acid (FA) metabolism.^30^

Th17 cells, characterized by their secretion of the cytokines IL‐17A and IL‐17F, follow a unique pathway of differentiation and development. Under the influence of transforming growth factor‐b (TGF‐b) and IL‐6, naïve CD4^+^ T cells commit to the initial stage of Th17 differentiation. Upon stimulation by endogenous or exogenous pathogen‐associated signals, dendritic cells (DCs) and tissue‐resident macrophages secrete IL‐23. The binding of IL‐23 to its receptor, IL‐23R, activates the Janus kinase 2 (JAK2) and tyrosine (Tyr) kinase 2 (TYK2), activating STAT3. This activation leads to the upregulation of the retinoic acid receptor‐related orphan receptor γ‐t (RORγt) transcription factor, which acts as the “master regulator” of Th17 cell differentiation.^31^ RORγt is a nuclear receptor that requires interaction with lipid ligands to achieve transactivation.^32^ The differentiation of Th17 cells relies on the de novo synthesis of FA.^33^ Culturing Th17 cells with IL‐23 and IL‐1β leads to increased inflammatory phenotypes and upregulation of genes related to FA biosynthesis. Acetyl‐CoA carboxylase 1 (ACC1) can regulate the biosynthesis of FAs and thereby control the differentiation of Th17 cells. The nuclear receptor PPAR‐γ can influence the lipid metabolism of cells, thereby affecting the differentiation and function of Th17 cells.^34^

In response to TCR signaling, CD4^+^ SP thymocytes differentiate into CD25^+^Foxp3^−^ CD4SP T regulatory cell precursors. The engagement of TCR–CD28 can activate the NF‐κB and PI3K–Akt signaling pathways. Among the NF‐κB family members, c‐Rel is essential in developing thymus‐derived Foxp3^+^ Treg cells, also known as tTreg cells.^35^ Glutamine deprivation has been demonstrated to enhance the expression of the transcription factor Foxp3 in Treg cells. Besides, the inhibition of glutamate conversion to α‐ketoglutarate has been shown to promote the differentiation of Th17 cells towards Treg cells through methylation of the Foxp3 locus.^36^ However, the activation of the PI3K–Akt signaling pathway has been demonstrated to inhibit the differentiation of tTreg cells. Thus, the differentiation and maturation of tTreg cells are subject to the precise regulation of NF‐κB and PI3K–Akt signaling pathways triggered by antigenic stimulation. Following this, CD25^+^Foxp3^−^ CD4SP Treg precursors, upon stimulation by the common γ‐chain cytokine IL‐2 and other signals, initiate the transcriptional mechanisms that govern the expression of the Foxp3 gene, culminating in the formation of tTreg cells. Furthermore, transcription factors such as STAT5 and the orphan nuclear receptors of the nuclear receptor 4a (Nr4a) family—comprising Nr4a1, Nr4a2, and Nr4a3—also influence the differentiation of tTreg cells to vary extents.^35^ The expression levels of Glut1 on the cell surface of Th1, Th2, and Th17 cells are higher than those on Treg, yet Glut1 is essential for the differentiation and development of CD4^+^ Treg cells. Treg cells exhibit lower glycolysis rates and higher FAO rates, consistent with their higher levels of phosphorylated serine/threonine kinase AMP‐activated protein kinase (AMPK). AMPK contributes to the stability and function of Treg cells by suppressing Glut1 expression, promoting FAO, and increasing the expression of Foxp3.^33^

Emerging research posits that CD8 T cells with autoreactivity in the thymus do not necessarily undergo direct apoptosis following positive and negative selection. Instead, signals mediated by TCR induce a decrease in the expression of the transcriptional repressor Gfi1. This downregulation of Gfi1 activates the sphingosine‐1‐phosphate receptor‐1 (S1P1) on the surface of immature thymocytes. Consequently, these immature CD8 T cells are prematurely ejected from the thymus into the periphery. There, they are believed to undergo further maturation, eventually developing into mature CD8 T cells that have acquired self‐tolerance.^37^ This paradigm underscores the intricate regulatory mechanisms within the thymus, highlighting the dynamic interplay between TCR signaling, transcriptional regulation, and cellular egress. It provides a nuanced view of how the immune system refines its T cell repertoire, balancing eliminating potentially self‐reactive cells with maintaining a diverse and functional immune response. Naive CD8 T cells reside in a metabolically quiescent state, primarily fueled by FAO. Upon antigenic stimulation of CD8 T cells, the costimulatory molecule CD28 becomes activated, leading to increased expression of Glut1, lactate dehydrogenase A, and hexokinase, which enhances glycolysis and directs metabolized glucose towards the synthesis of nucleotides and serine. The activation of CD28 also results in decreased expression of carnitine palmitoyltransferase 1A, coupled with the mTOR‐regulated sterol regulatory element‐binding proteins, which leads to increased FA synthesis.^38^

### 2.2 Function regulation of DC by immunometabolism pathway

DCs, in their specialized role as APCs, connect the innate and adaptive arms of immunity.^39^, ^40^ As sentinels of the immune response, they are adept at capturing, processing, and presenting antigens to T cells, thus initiating and shaping adaptive immune reactions. DCs originate from HSC, and their differentiation process is modulated by the hematopoietic growth factor Fms‐like TYK3 ligand (FLT3L).^41^ FLT3L is pivotal in regulating HSC status by binding to its cognate receptor FLT3. This interaction induces receptor homodimerization and subsequent autophosphorylation, activating downstream signaling cascades. FLT3L engagement activates the PI3K–Akt pathway, which is instrumental in promoting cell survival, proliferation, and metabolism. Additionally, FLT3L signaling intersects with the Ras/MAPK pathway, known for its role in cell growth and differentiation. By modulating these critical pathways, FLT3L influences the maintenance, self‐renewal, and differentiation potential of HSCs, thereby shaping the hematopoietic landscape.^42^, ^43^, ^44^, ^45^ FLT3L stimulates the expression of nutrient transport proteins CD71, CD98, and Glut1, enhancing dextran uptake by conventional type 1 DCs (cDC1).^46^ When aerobic glycolysis, which is associated with anabolic metabolism, is inhibited by the inhibitor 2‐DG, the cell proliferation induced by Flt3L is blocked by 2‐DG in a dose‐dependent manner, indicating that the development of DCs is dependent on aerobic glycolysis.^47^ When bone marrow‐derived DCs are cultured in the presence of FLT3L (FL‐DCs), there is an upregulation of the expression of key surface molecules such as CD80, CD86, and MHC II, which are critical for effective T cell activation. Upon encountering an infection, FL‐DCs exhibit an enhanced capacity to produce many proinflammatory cytokines, including IL‐6, IL‐12, IL‐23, IFN‐γ, and tumor necrosis factor‐α (TNF‐α).^48^ Research has demonstrated that FL‐DCs exhibit superior migratory capacity when administered via subcutaneous injection, effectively trafficking to the draining lymph nodes. This enhanced migration is a critical aspect of their function in initiating immune responses at peripheral sites. From a cellular perspective, FL‐DCs possess characteristics that resemble those of steady‐state resident DCs more closely.^49^

Granulocyte‐macrophage colony‐stimulating factor (GM‐CSF) is recognized as a pivotal regulatory cytokine for DC development under both homeostatic and inflammatory conditions, serving as a central mediator of tissue inflammation.^50^ Under homeostatic conditions, the expression of GM‐CSF is typically maintained at low levels, ensuring a baseline level of DC function. However, upon encountering infection and inflammation, the expression of GM‐CSF escalates, endowing DCs with enhanced capabilities for rapid antigen uptake and cross‐presentation.^51^ Upon binding to its receptor, GM‐CSF activates the cytoplasmic Tyr kinase JAK2, triggering the JAK/STAT pathway, MAPK pathway, and PI3K pathway was also activated. These constitute key signaling modules that regulate diverse cellular processes such as proliferation, survival, and differentiation. Furthermore, GM‐CSF activation leads to the canonical NF‐κB signaling cascade, a central component in immune and inflammatory responses. The activation of these pathways by GM‐CSF is essential for the maturation and functional specialization of various DC subsets, thereby facilitating their role in antigen presentation and the modulation of adaptive immunity.^50^ DCs cultured with GM‐CSF exhibit a distinct cytokine profile when stimulated with Toll‐like receptor (TLR) agonists. Compared with FL‐DCs, GM‐CSF‐cultured DCs produce a unique array of cytokines, including TNF‐α, IL‐10, and the chemokine CCL2.^49^ This differential cytokine production is reflective of the cells' activation state and their role in shaping the immune response. The secretion of TNF‐α and IL‐10 suggests a role in both proinflammatory and regulatory processes, while CCL2 contributes to leukocyte chemotaxis and inflammation. GM‐CSF can also augment dextran uptake by DCs and effectively sustain the vitality of cDCs.^46^, ^52^ Under the stimulation of GM‐CSF and IL‐4 in vitro, PPAR‐γ, which plays a crucial role in FAO, is significantly upregulated in DCs. The mRNA levels of PPAR‐γ target genes, including fatty‐acid‐binding protein‐4, adipose differentiation‐related protein, and apolipoprotein E, are increased, modulating the differentiation of DC subpopulations.^47^

In plasmacytoid DCs (pDCs), the induction of the JAK2–STAT5 signaling pathway by IL‐3 culminates in generating transport proteins SLC7A5 and SLC3A2. These transport proteins, involved in the uptake of the essential amino acid leucine, work in concert with TLR activation to engage mTORC1. The engagement of transport proteins and TLR signaling leads to the activation of mTORC1, a key instigator of anabolic metabolism, which can promote a suite of synthetic metabolic activities associated with increased glycolysis, thereby facilitating the production of cytokines and chemokines by this specific subset of DCs.^53^, ^54^, ^55^

### 2.3 Immunometabolism modulation in macrophage polarization and function

Macrophages are a class of immunoeffector cells with remarkable plasticity, ubiquitously present in all tissues of living organisms. They possess the innate ability to rapidly alter their functional characteristics in response to various stimuli, thereby playing pivotal roles in both innate and adaptive immune responses.^56^ Cytokines such as IFN‐γ, TNF‐α, IL‐1, IL‐4, and IL‐10 are crucial in orchestrating the polarization and metabolic reprogramming of macrophages. IFN‐γ promotes the polarization of M1‐type macrophages by activating signaling pathways such as JAK/STAT and NF‐κB.^57^, ^58^ Additionally, lipopolysaccharide (LPS) can also induce M1 polarization in macrophages, a process that is typically accompanied by an increase in aerobic glycolysis. M1 macrophages, aerobic glycolysis is coupled to the oxidative PPP to generate NADPH. This NADPH is subsequently utilized by NADPH oxidases to produce reactive oxygen species (ROS). The generation of ROS is pivotal for activating the NLRP3 inflammasome, which is crucial for the phagocytic activity of M1 macrophages.^58^, ^59^ The interplay between aerobic glycolysis, NADPH production, and ROS generation is integral to the proinflammatory functions of M1 macrophages, highlighting the critical role of metabolic pathways in shaping immune responses.

IL‐4 and IL‐13 are pivotal cytokines that promote the polarization of M2 macrophages through the activation of the JAK/STAT signaling pathway.^60^ The polarization of M2 macrophages is also contingent upon FAO. Research has demonstrated that M2 macrophages utilize the uptake and oxidation of FAs to fuel the TCA cycle, subsequently providing energy for oxidative phosphorylation (OxPhos). The inhibition of FAO results in the suppression of M2 macrophage activation induced by IL‐4, underscoring the critical role of FAO in their metabolic programming and functional activation. In addition to FAO, glycolysis is also essential for the biosynthesis of cytokines in M2 macrophages.^61^, ^62^ This metabolic pathway not only generates ATP but also serves as a source of precursors for anabolic processes, highlighting the dual importance of glycolysis in both energy production and the inflammatory response of M2 macrophages.

### 2.4 Immunometabolism regulation in natural killer cell development and cytotoxicity

Natural killer (NK) cells originate from HSC and are integral components of the innate immune system, characterized by their ability to exert nonspecific cytotoxic effects against target cells. Resting NK cells maintain a low level of glycolysis and OxPhos, a metabolic rate sufficient to produce IFN‐γ, thereby sustaining acute NK cell responses. Notably, inhibiting either OxPhos or glycolysis can impact the generation of IFN‐γ.^63^ IL‐15 plays a pivotal role in the differentiation and development of NK cells, serving as a critical cytokine in their maturation process. Upon binding to the IL‐15Rβ/γ complex, IL‐15 activates JAK1 and JAK3, promoting the dimerization or tetramerization of STAT5. This signaling cascade leads to the transcriptional activation of the target gene *Mcl1*, resulting in the production of the antiapoptotic protein MCL1. MCL1 functions to sequester proapoptotic proteins such as BIM and NOXA, thereby sustaining the continuous survival of NK cells. Conversely, the withdrawal of IL‐15 signaling can precipitate apoptosis in NK cells. Furthermore, the enhancement of IL‐15 signaling augments the expression of key effector molecules within NK cells, including IFN‐γ, perforin, and granzymes. This upregulation not only bolsters the cytotoxic potential of NK cells but also stimulates their proliferation and differentiation.^64^ Activated NK cells under the stimulation of cytokines, such as IL‐2, experience an increase in mTORC1 activity, which leads to elevated expression of nutrient transport proteins and glycolytic enzymes. When the activity of mTORC1 is inhibited, the glycolysis in NK cells decreases. It is noteworthy that the increase in glycolysis in NK cells stimulated by IL‐15 does not require the active involvement of mTORC1. Furthermore, mTORC2 restricts the activity of mTORC1 by inhibiting the expression of the amino acid transporter SLC7A5, which is mediated by STAT5.^63^

IL‐2, a cytokine belonging to the same 4‐α‐helix bundle family as IL‐15, rapidly enhances NK cells’ ability to bind to their target cells. This effect is particularly notable in augmenting NK cells’ reactivity against weak target cells, thereby strengthening their overall cytotoxic function. However, IL‐2 does not exert an influence on the formation and cytolytic activity of NK cells against strong target ligands, which suggests a nuanced role in modulating the responsiveness of NK cells based on the strength of target cell recognition.^65^ NK cells activated by IL‐2 primarily derive their energy from glucose metabolism, and they rely on the activity of mTORC1 to enhance glycolysis levels.^63^

Traditionally, NK cells have been considered to lack memory capabilities. However, recent findings challenge this notion. Under the combined stimulation of cytokines IL‐12, IL‐15, and IL‐18, NK cells exhibit heightened metabolic activity and induce a “memory‐like” phenotype. This subset of NK cells, after brief activation, can mount a more robust response upon subsequent cytokine re‐stimulation, characterized by the production of increased levels of IFN‐γ.^66^

## 3 HOMEOSTATIC REGULATION OF IMMUNOMETABOLISM

The homeostatic regulation of immunometabolism is an intricate and sophisticated process that ensures immune cells operate at peak efficiency without damaging the host organism. The regulation encompasses a complex array of signaling pathways and metabolic sensors attuned to the ebb and flow of nutrient availability, energy requirements, and cellular stressors, thereby maintaining a delicate balance essential for immune system function and overall health.

### 3.1 Maintenance of metabolic balance in immune cells

Metabolic pathways are not only crucial for energy production but also serve as a signaling hub that integrates extracellular signals with cellular responses. From monitoring and defending against pathogens to resolving inflammation and tissue repair, immune cells require the delicate regulation of metabolic activities to perform their diverse functions and maintain systemic homeostasis.

Within the immune cell repertoire, the mTOR serves as a central modulator of cellular growth and metabolic pathways. Upon activation, the mTOR pathway orchestrates enhancing anabolic processes, which are crucial for facilitating T lymphocytes’ clonal expansion and developmental trajectory.^67^ Autophagy is a critical degradation and recycling process within immune cells, essential for maintaining their homeostasis and functionality. In T cell‐mediated immune responses, autophagy exerts its effects through various mechanisms. In conditions of nutrient abundance, the activation of mTORC1 typically inhibits autophagy.^68^ However, under various environmental stresses, such as nutrient deprivation, absence of growth factors, and hypoxia, mTORC1 is either not activated or its activity is subdued. This leads to the induction of autophagy through multiple pathways, which helps cells maintain sufficient nutrition and energy supply to ensure their survival.^69^ Glucose starvation can activate AMPK by increasing the relative levels of AMP or ADP to ATP, thereby triggering autophagy, or it can initiate autophagy by enhancing amino acid catabolism, leading to an accumulation of ammonia.^70^ Autophagy is a crucial mechanism for promoting cellular homeostasis, adapting to metabolic demands within the body, and maintaining metabolic balance within cells. This intricate process is vital for the overall health and proper functioning of cells, ensuring they can meet the energetic and material needs of the organism.

SREBP plays a crucial role in the metabolic reprogramming induced by the activation of CD8^+^ T cells. The absence of SREBP not only suppresses lipid synthesis but also markedly inhibits the proliferation of T cells.^34^ SREBP‐1a and ‐2 are the principal isoforms of SREBP, which activate the synthesis of FAs and cholesterol, respectively. The activation of SREBP by mTORC1 is mediated through regulating cholesterol transport. When mTORC1 is highly active, it suppresses autophagy and inhibits the transport of cholesterol from lysosomes to the endoplasmic reticulum, leading to reduced cholesterol levels in the endoplasmic reticulum and consequently promoting the activation of SREBP‐2 and the expression of genes involved in cholesterol synthesis. In contrast, when mTORC1 activity is low, autophagy is induced, and cholesterol is transported to lysosomes through two membrane transport pathways, resulting in increased endoplasmic reticulum cholesterol levels, which suppresses the activation of SREBP‐2 and inhibits cholesterol synthesis.^71^

Liver kinase B1 (LKB1) plays a crucial role in maintaining T cell homeostasis by regulating mTORC1 through the phosphorylation of phosphatase and tensin homolog (PTEN). In the absence of LKB1, T cells exhibit a metabolic shift characterized by increased glycolysis mediated by mTORC1–HIF1α, which subsequently influences the differentiation of Th1 and Th17 cells, thereby contributing to the development of inflammation.^72^

### 3.2 Influence of the microenvironment on immune cell metabolism

The metabolic activities of immune cells are fundamental to their functional execution,^6^ with these activities being significantly influenced by the microenvironment in which they reside. Nutrients, oxygen levels, and cytokines within the microenvironment are pivotal in sculpting the metabolic pathways of immune cells.^73^ These factors subsequently dictate the survival, proliferation, and functional performance of immune cells.

Under homeostatic conditions, immune cells meticulously regulate their metabolic pathways to maintain their functionality and self‐renewal capabilities. Quiescent T cells predominantly rely on the breakdown of glucose, and amino acids to actively inhibit the aberrant activation of mTORC1. Following activation, T cells escalate their glucose metabolism and enhance the catabolism of glutamine to foster cellular growth, division, and differentiation.^74^ However, this delicate metabolic balance is often disrupted in the tumor microenvironment. Tumor cells, through enhanced aerobic glycolysis, consume a significant amount of glucose, leading to reduced glucose levels in the tumor interstitial fluid, which in turn affects the survival and functionality of T cells.^75^ In the hypoxic or glucose‐deprived cancer microenvironments, CD8^+^ tumor‐infiltrating T lymphocytes (TILs) can augment their uptake and catabolism of FAs to generate more energy and restore their antitumor capabilities.^76^ Because Treg cells are less dependent on glucose metabolism, they are more capable of adapting to the tumor microenvironment, characterized by high lactate and low glucose levels. In highly glycolytic tumors, Treg cells absorb lactate through the monocarboxylate transporter 1 (MCT1), which increases the expression of PD‐1 on Treg cells, thereby affecting the curative effect.^33^ Tumor cells and tumor‐associated macrophages (TAMs) that highly express PD‐L1 can impair the PI3K/AKT/mTOR pathway, directly affecting glycolysis. Additionally, PD‐L1 can suppress glutaminolysis, blocking energy supply to CD8^+^ T cells derived from glutamate metabolism. Furthermore, PD‐L1 may also activate STAT3, promoting FAO in CD8^+^ T cells, thereby harming these cells and inhibiting antitumor immunity mediated by CD8^+^ T cells.^77^ In tumors with high glycolysis, cancer cells produce a large amount of lactate through an enhanced glycolytic process. This lactate can affect the function of pyruvate carboxylase, thereby impacting the TCA cycle, and ultimately influencing the cytotoxic killing function of CD8^+^ T cells, leading to tumor immune evasion. However, pyruvate dehydrogenase can inhibit this process, allowing CD8^+^ T cells to maintain their cytotoxicity and overcome the lactate‐rich TME.^78^ An increase in the extracellular asparagine level facilitates the uptake of Asn by T cells through the SLC1A5‐mediated antiporter mechanism. This process leads to the direct binding of Asn to lymphocyte‐specific protein Tyr kinase (LCK), inducing Tyr394 phosphorylation and subsequently activating LCK signaling. As a result, the activation of CD8^+^ T cells is enhanced, bolstering their antitumor activity in vivo.^79^

In mice infected with various pathogens such as vaccinia virus, Listeria monocytogenes, and lymphocytic choriomeningitis virus, researchers have observed that IL‐7 and IL‐15 can promote the expression of the antiapoptotic protein Bcl‐2, thereby facilitating the homeostatic turnover of memory T cells. Furthermore, IL‐7 and IL‐15 can also induce the expression of the glycerol channel protein aquaporin‐9 in CD8^+^ T cells, promoting the esterification of FAs and the formation of triglycerides, allowing memory T cells to maintain a stable and long‐term energy supply, thus extending the lifespan of CD8^+^ T cells.^33^

Primary splenic DCs or GM‐CSF BMDCs that ingest excessive FAs may compromise their ability to stimulate T cell proliferation, resulting in DC dysfunction.^80^ In TME, DCs acquire exogenous lipids, which diminish their immunogenicity, thereby facilitating the immune evasion of tumor cells.^81^ In contrast, liver DCs with higher lipid content can more effectively activate proinflammatory T cells, NK T cells, and NK cells. It is the distinct microenvironments that shape the varying lipid requirements of DCs.^80^

## 4 IMMUNOMETABOLIC CHECKPOINTS

Immunometabolic checkpoints primarily modulate immune cell metabolism, thereby enhancing or inhibiting immune responses through signaling pathways that impact the activation, differentiation, and effector functions of immune cells. This regulatory role is crucial for the survival and functionality of immune cells under various physiological and pathological conditions.

### 4.1 Immunometabolic checkpoints on T cells

#### 4.1.1 CD38

The CD38 molecule, a singular 300‐amino‐acid structure, presents an “L”‐shaped conformation and encompasses two distinct domains. As a single‐stranded glycoprotein with a solitary transmembrane segment, it is strategically positioned on the cell surface and within intracellular compartments such as the endoplasmic reticulum, nuclear envelope, and mitochondria. Predominantly exhibiting type II membrane orientation, CD38 directs its catalytic site outward, establishing it as the principal nicotinamide adenine dinucleotide nucleosidase (NADase) in mammals.^82^, ^83^, ^84^, ^85^ Initially recognized as a thymocyte marker in 1980, CD38's expression is notably induced by IFN‐γ, with levels correlating to the concentration and duration of IFN‐γ exposure. Other inducers include all‐trans retinoic acid, IFN‐β, and LPS, while IL‐2 exerts a milder effect. In contrast, TNF‐α and GM‐CSF show no significant influence on CD38 expression.^86^, ^87^, ^88^

CD38's enzymatic prowess lies in its ability to hydrolyze NAD into cyclic adenosine diphosphate ribose (cADPR) or ADPR and facilitate the exchange of bases in the hydrolysis of NADP into NADDP.^84^, ^89^, ^90^ These molecules serve as second messengers, instigating the release of Ca^2+^ and thereby modulating cellular growth, insulin secretion, T cell activation, and a suite of physiological processes. Notably, cADPR, upon various stimuli, triggers Ca^2+^ release through RYR2 and/or RYR3, activating store‐operated calcium entry in T cells, while intracellular ADPR governs calcium entry into LTRPC2‐expressing cells, and NAADP‐induced Ca^2+^ is implicated in spontaneous diastolic Ca^2+^ transients.^89^, ^91^, ^92^, ^93^, ^94^

A key catalytic residue, Glu‐226, within CD38, together with strong hydrogen bond interactions, sequesters cADPR at the active site, facilitating its biological role.^95^ As a cell surface molecule engaged in recognition and signal transduction, CD38 can interact with external molecules and other surface antigens, endowing it with surface receptor capabilities. Ligands such as CD31, agonistic monoclonal antibodies, and G protein‐coupled receptors can bind to CD38, initiating transmembrane signaling cascades that regulate the body's immune state.^89^, ^96^

Overexpression of CD38 has been noted in multiple myeloma (MM),^97^ chronic lymphocytic leukemia (CLL),^98^, ^99^ acute lymphoblastic leukemia (ALL),^100^ Waldenstrom's macroglobulinemia,^101^ B‐cell non‐Hodgkin lymphoma,^102^ and other malignancies, highlighting its clinical significance.

During the process of thymic selection, the interaction between CD38 and CD28 is instrumental in inducing apoptosis in most DP thymocytes, a critical step in preserving immune homeostasis.^103^ While CD38 has been shown to activate T cells, stimulate proliferation, and mediate the production of cytokines such as IL‐1, IL‐6, IL‐10, IFN‐γ, TNF‐α, and granzyme B, enhancing T cell function, it has also been implicated in inhibiting T cell function and advancing disease progression.^104^, ^105^ other studies have also shown that CD38 inhibits T cell function and promotes disease progression. In the context of human immunodeficiency virus (HIV), hepatitis B virus, and other infections, elevated levels of CD38^+^CD8^+^ T cells are tightly linked to disease progression and viral load.^106^, ^107^ It is hypothesized that post‐HIV infection, overexpression of CD38 may deplete NAD in CD4^+^ T cells, leading to a chronic Warburg effect and mitochondrial dysfunction. Metabolites of CD38, including ADPR and cADPR, are suspected of disrupting mitochondrial integrity by increasing cytoplasmic Ca^2+^ levels, impairing the viability and regenerative capacity of CD4^+^ T cells. This depletion is exacerbated by the proliferation of CD8^+^ T cells expressing CD38, contributing to systemic NAD reduction and promoting HIV disease progression.^108^ CD38‐expressing CD8^+^ T cells exhibit mitochondrial dysfunction, impairing IFN‐γ and TNFα secretion and leading to an exhaustion phenotype.^109^ CD38's role in the production of the immunosuppressive molecule adenosine, via the CD38/CD203a/CD73 extracellular enzyme pathway, further modulates immune responses by binding to adenosine receptors on CD8 T cells, inhibiting their proliferation and facilitating tumor immune escape.^110^, ^111^ IL‐15 has also been shown to enhance suppressive function and impact the viability and expansion of CD35^+^CD8^+^ T cells. In vitro studies suggest that CD38^+^CD8^+^ T cells can broadly inhibit the proliferation of CD4 effector T cells through IFN‐γ.^112^ Further investigation is warranted to fully understand the implications of CD38 surface expression on intracellular NAD levels.

#### 4.1.2 IDO1/2 and TDO

IDO1 and IDO2, along with TDO, act as the primary rate‐limiting enzymes in the catabolism of tryptophan (Trp) via the kynurenine (Kyn) pathway. IDO1 and IDO2 are two distinct isoforms, with IDO1 characterized by a heme monomer nestled between a pair of α‐helical domains,^113^ and IDO2 exhibiting greater substrate specificity for Trp with comparatively lower catalytic activity.^114^ TDO, a tetrameric enzyme, is versatile in its substrate specificity, catalyzing not only Trp but also its fluorinated analogs.^115^ IDO1 is predominantly found in glial cells, neurons, microglia, DCs, monocytes, and macrophages,^116^ whereas IDO2 is primarily expressed in certain tumor cells.^114^, ^116^ Additionally, IDO2 is mainly detected in certain tumor cells and is constitutively present in the liver, placenta, and central nervous system.^117^ TDO's primary site of expression is the liver.^118^ The expression of IDO1 is primarily triggered by IFNs, TNF‐α, LPS, and IL‐10,^119^, ^120^ whereas IDO2 expression is induced by the aryl hydrocarbon receptor (AHR).^121^ TDO expression can be stimulated by glucocorticoids.^122^

Functionally, both IDO and TDO cleave the C2‐C3 bond of indole ring of Trp introducing an oxygen molecule to form N‐formyl‐Kyn, which rapidly and spontaneously transforms into Kyn.^123^ Kyn is further processed by a series of enzymes into immunosuppressive metabolites, including kynurenic acid (KYNA), xanthurenic acid (XA), 3‐hydroxyanthranilate (3‐HAA), and quinolinic acid (QA), ultimately yielding NAD, a vital coenzyme within the body.^118^, ^124^, ^125^, ^126^, ^127^

**FIGURE 2:**
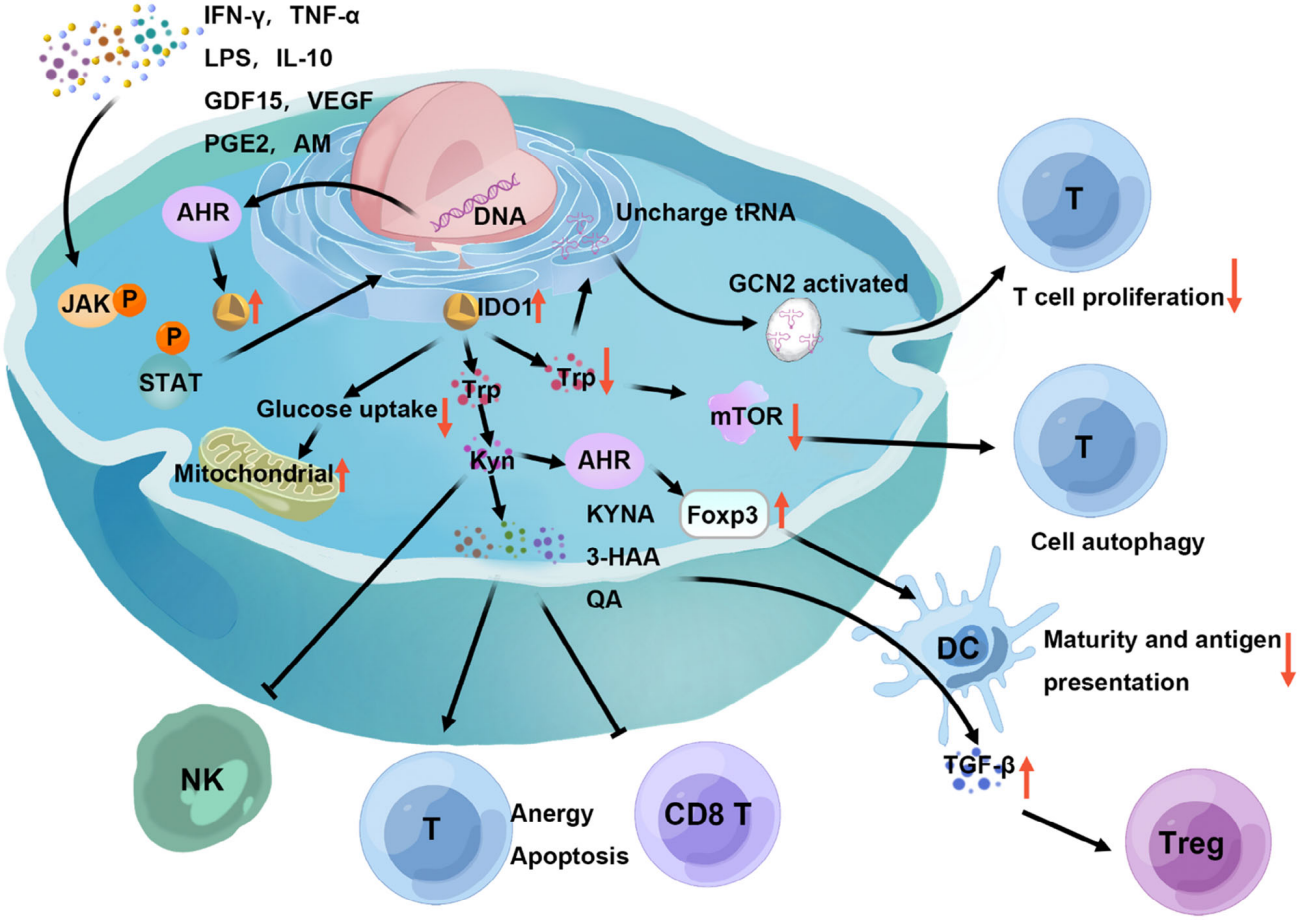
The activation of IDO1 and its role in immunosuppression. IDO1 is induced by a spectrum of cytokines, and it is instrumental in fostering immune tolerance via three distinct mechanisms: modulation of cellular metabolism, accumulation of tryptophan metabolites, and the depletion of tryptophan.

IDO1's immunosuppressive effects are exerted through three principal mechanisms: “Trp depletion,” “accumulation of Trp metabolites,^128^” and “cell metabolism involvement (Figure 2).”^129^ Initially, cells expressing IDO1 consume Trp aggressively. In Trp‐sufficient conditions, the activated mTOR phosphorylates ribosomal S6 kinase, which in turn phosphorylates ribosomal protein S6, promoting cellular growth, proliferation, and protein synthesis. Conversely, Trp deficiency triggers the storage of uncharged Trp‐tRNA, activating the Trp‐sensing kinase general control nonderepressible 2 (GCN2) and leading to the phosphorylation of eukaryotic initiation factor 2 alpha. This phosphorylation event disrupts mRNA translation and curbs protein synthesis, resulting in T cell cycle arrest and a consequential impact on T cell proliferation. Additionally, Trp scarcity impairs mTOR pathway activity and induces mTOR‐dependent autophagy.^130^, ^131^, ^132^, ^133^, ^134^, ^135^ Second, IDO1 enzymatically degrades Trp into Kyn, which activates AHR and fosters the expression of Foxp3 in T cells, thus driving the differentiation of CD4^+^ T cells into Tregs.^136^, ^137^, ^138^ This process also attracts TAMs,^139^ modulates DC maturation,^140^ and creates an immunosuppressive tumor microenvironment conducive to cancer cell evasion, metastasis, and drug resistance development. Kyn has been shown to induce NK cell apoptosis via a ROS‐mediated pathway and impairs NK cell function by dampening the surface expression of activating receptors NKp46 and NK group 2D (NKG2D).^141^ AHR also regulates IDO1 expression.^142^ Kyn is further metabolized into KYNA, 3‐HAA, QA, and other catabolic products that bind to immune cell receptors, promoting immune tolerance and dampening T cell responses, leading to T cell anergy, apoptosis, Treg and Th17 cell proliferation, and alterations in Th1/Th2 balance.^117^

Lastly, IDO1 diminishes glucose uptake in activated lymphocytes, enhances mitochondrial function, curbs aerobic glycolysis, and stimulates the production of the immunosuppressive cytokine IL‐10 by lymphocytes. This metabolic interference by IDO1 further exerts regulatory effects on the immune response. Studies have revealed a synergistic interaction between “Trp depletion” and “accumulation of Trp metabolites” within the IDO1 regulatory network, culminating in an amplified immunosuppressive effect that significantly influences the body's immune response.^129^

The expression of IDO2 curtails the proliferation of CD4^+^ and CD8^+^T cells,^143^ and impedes the infiltration of CD4^+^ T cells into tumor sites.^144^ IDO2, triggered by LPS, directly stimulates cytokine production by modulating the IL‐6/stat3 signaling pathway, thereby influencing T cell function.^145^ Studies have further revealed that the differentiation of Tregs induced by IDO1 significantly depends on IDO2.^127^ Additionally, TDO suppresses T cell responses by generating the tryptophan metabolite Kyn.^146^

#### 4.1.3 Arginase

Arginine, a conditionally essential amino acid, is indispensable for the synthesis of other amino acids, including proline and glutamate, as well as for the production of polyamines, urea, and guanidine. It plays a pivotal role in regulating T‐cell receptor expression and the development of immune memory.^147^ Arginase, a manganese‐dependent metalloenzyme, catalyzes the final step of the urea cycle, converting l‐arginine into l‐ornithine and urea. Two isoforms of arginase exist: arginase1, predominantly a cytoplasmic enzyme expressed in the liver, and arginase2, which is primarily mitochondrial and highly expressed in the kidney, small intestine, and brain.^148^, ^149^ Despite being encoded by different genes, both isoforms exhibit similar enzymatic activities.

Arginase1 mediates immunosuppression through two primary mechanisms that render lymphocytes unresponsive to antigenic challenges. First, arginase1 depletes arginine, leading to reduced TCR membrane expression and loss of CD3ζ chain, which disrupts T cell activation and proliferation. This depletion also affects the secretion of IFN‐γ, IL‐5, and IL‐10, and inhibits IL‐2 production. Second, l‐ornithine, a byproduct of arginase1 activity, is integral to the synthesis of polyamines, essential for the activation and proliferation of CD4 and CD8 T lymphocytes in mice.^150^, ^151^, ^152^

Macrophages and myeloid‐derived suppressor cells (MDSCs) upregulate arginase1 expression in response to IL‐4 and IFN‐γ,^149^ while human granulocytes constitutively express arginase1.^153^ The key transcription factors driving arginase1 expression are STAT 6 and STAT3.^153^, ^154^ In hepatitis C virus (HCV) research, it was found that HCV‐induced MDSCs could impair mTOR activation, thereby suppressing IFN‐γ production in NK cells. Arginase inhibitors can reverse this suppression, restoring IFN‐γ secretion.^155^ Arginase2 operates similarly to arginase1 in inducing immunosuppression and is found in CD8^+^ T cells, where it exerts a regulatory function that suppresses their ability to modulate tumor growth.^156^

Moreover, MDSCs expressing arginase1 contribute to the immune system's modulation by enhancing the production of ROS and inducible nitric oxide synthase.^157^ M2 TAMs, tolerogenic DCs, and Tregs expressing arginase1 can suppress antitumor immune responses by limiting T cell arginine uptake, leading to T cell depletion at the tumor site and fostering tumor progression.^158^ STAT1 signaling regulates arginase1 activity in TAMs, which in turn affects T cell responses and contributes to the tumor's ability to evade the immune system.^159^

#### 4.1.4 Histone deacetylase 6

The HDACs, a diverse group of enzymes, are classified into four main classes, with HDAC6 being a standout member. Class I includes HDAC1, HDAC2, HDAC3, and HDAC8, while Class II is bifurcated into IIa, comprising HDAC4, HDAC5, HDAC7, and HDAC9, and IIb, which includes HDAC6 and HDAC10. Class III is predominantly made up of the Sirtuin family (Sirtuins 1–7), and Class IV is home to a single member, HDAC11.^5^ HDAC6, the largest of the HDAC family, is unique in its possession of two fully functional catalytic domains.^5^, ^160^ This zinc‐dependent enzyme is predominantly cytoplasmic and is highly conserved across species. Its catalytic domain 1 (CD1) exhibits both deacetylase and ubiquitin E3 ligase activities, whereas CD2 serves as a versatile deacetylation domain.^5^

HDAC6 is known for its role in regulating the acetylation of nonhistone proteins,^161^ interacting with a variety of cytoplasmic proteins such as tubulin, heat shock protein (HSP90), Peroxiredoxins I and II, and Survivin. These interactions are crucial in mediating neurodegenerative processes, neurological dysfunction, seizure promotion, modulation of host antiviral responses, and cancer progression.^5^, ^162^, ^163^ HDAC6's expression is notably increased in Tregs, where it plays an essential role in the induction of Foxp3. Inhibition of HDAC6 leads to a loss of characteristic features in induced Tregs (iTregs), resulting in altered Treg function.^164^

HDAC6 also interacts with the forkhead box protein O1 (FoxO1) in the cytoplasm of Th17 cells, deacetylating it at the K242 site and thereby inhibiting the transcription factor RoRγt. This action diminishes the mRNA expression of IL‐17A, IL‐17F, IL‐23R, IL‐22, and IL‐1R, as well as the secretion of IL‐17A and IL‐17F. Additionally, it curbs the expression of FoxO1 target genes, including CCR7, lymphoid enhancer‐binding factor 1, and transcription factor 7. The genetic deletion of HDAC6 bolsters the responsiveness of CD8^+^ T cells and enhances their antitumor capabilities. In the context of the tumor microenvironment, Th17 cells lacking HDAC6 promote survival through the activation of IFN‐γ and granzyme B signaling pathways.^165^

#### 4.1.5 IL‐4 induced 1

IL4I1 is a glycosylated secretory protein that functions as an l‐amino acid metabolizing enzyme, utilizing dimeric flavine adenine dinucleotide as a cofactor. It is a member of the l‐amino‐acid oxidase family and is predominantly expressed in myeloid cells, DCs, and both T and B lymphocytes within the immune system. Induction of high IL4I1 expression in myeloid cells can be triggered by Type I and II IFNs, and IL‐4 is capable of inducing IL4I1 expression in B cells.^166^, ^167^, ^168^

IL4I1 catalyzes the oxidation and deamination of phenylalanine, resulting in the production of hydrogen peroxide (H_2_O_2_) and phenylpyruvate. The generated H_2_O_2_ can lead to a transient down‐regulation of the TCRζ chain, diminishing TCR signal transduction and consequently inhibiting T cell proliferation.^167^ Furthermore, IL4I1 facilitates the conversion of Tyr to hydroxyphenylpyruvic acid and Trp to indole‐3‐pyruvic acid (I3P). The metabolites KYNA and I3A, derived from I3P, can activate AHR, promote the differentiation of Tregs, and recruit MDSCs^169^ Additionally, 13P can suppress ferroptosis in tumor cells by neutralizing free radicals and activating antioxidant stress pathways, thus modulating immunosuppression.^168^

IL4I1 also has the capacity to directly decrease the stability of T‐DC immune synapses in vitro without compromising the phenotype or antigen‐presenting capabilities of DCs, thereby inhibiting T cell activation and limiting the differentiation of memory T cells.^170^ Moreover, IL4I1 can stimulate the expression of AHR target genes TIPARP and CYP1B1 in CD8^+^ T cells, contributing to the suppression of CD8^+^ T cell function and potentially aiding tumor progression.^169^

Beyond its metabolic pathways, IL4I1 can also nonenzymatically interfere with the phosphorylation of ZAP70, a key signal transduction molecule in TCR signaling, leading to impaired T cell synapse formation and ultimately hindering full T cell activation and proliferation.^171^ Clinically, the expression of IL4I1 has been inversely correlated with the survival rates of glioma patients.^169^ and has been linked to an unfavorable prognosis in melanoma.^172^ and thyroid cancer.^173^

### 4.2 Immunometabolic checkpoints on DCs

#### 4.2.1 Indoleamine 2,3‐dioxygenase 1

The upregulated expression of IDO in DCs is particularly significant, as it wields immunosuppressive effects that directly target APCs, fostering the emergence of regulatory DCs and indirectly modulating local T cells.^128^, ^174^, ^175^, ^176^ IDO's role is multifaceted; it curbs the secretion of the proinflammatory cytokine IL‐12 by DCs while simultaneously promoting the release of the anti‐inflammatory cytokine IL‐10. This duality induces T cell tolerance by suppressing Th1 cell differentiation and encouraging the differentiation of Tregs.^177^ IDO1's influence extends to pDCs, where it mitigates IL‐6 production through a GCN2‐dependent mechanism, thus preserving the suppressive phenotype of Tregs.^178^

IDO‐expressing DCs are capable of effectively curbing the proliferation of CD4^+^CD25^−^ T cells in vitro and expanding the population of CD4^+^CD25^+^Foxp3^+^ Tregs via the IDO–Kyn–AHR axis.^179^, ^180^ The metabolite Kyn, produced by IDO, and its derivatives have been recognized as AHR ligands capable of activating T cell AHR signaling, further bolstering Treg differentiation.^181^ Moreover, IDO‐expressing DCs can enhance the expression of cytotoxic T lymphocyte antigen 4 (CTLA‐4) and PD‐1 on Tregs.^182^ The interaction between CTLA‐4‐expressing Tregs and the B7 molecule on DCs induces IDO expression, suggesting a self‐regulatory network where CTLA‐4^+^ Tregs harness IDO to dampen T cell responses and instill tolerance.^183^, ^184^, ^185^, ^186^

IDO^+^ DC can induce apoptosis in both CD4^+^ T cells and CD8^+^ T cells, as well as significantly inhibit the activity of CD8^+^ T cells.^187^ In addition to T cells, IDO^+^ DC can also reduce B cell activation factor expression on mononuclear cells, thereby affecting B cell maturation and survival and resulting in a decrease in the number of plasma cells in the spleen and local lymph nodes.^188^

**FIGURE 3:**
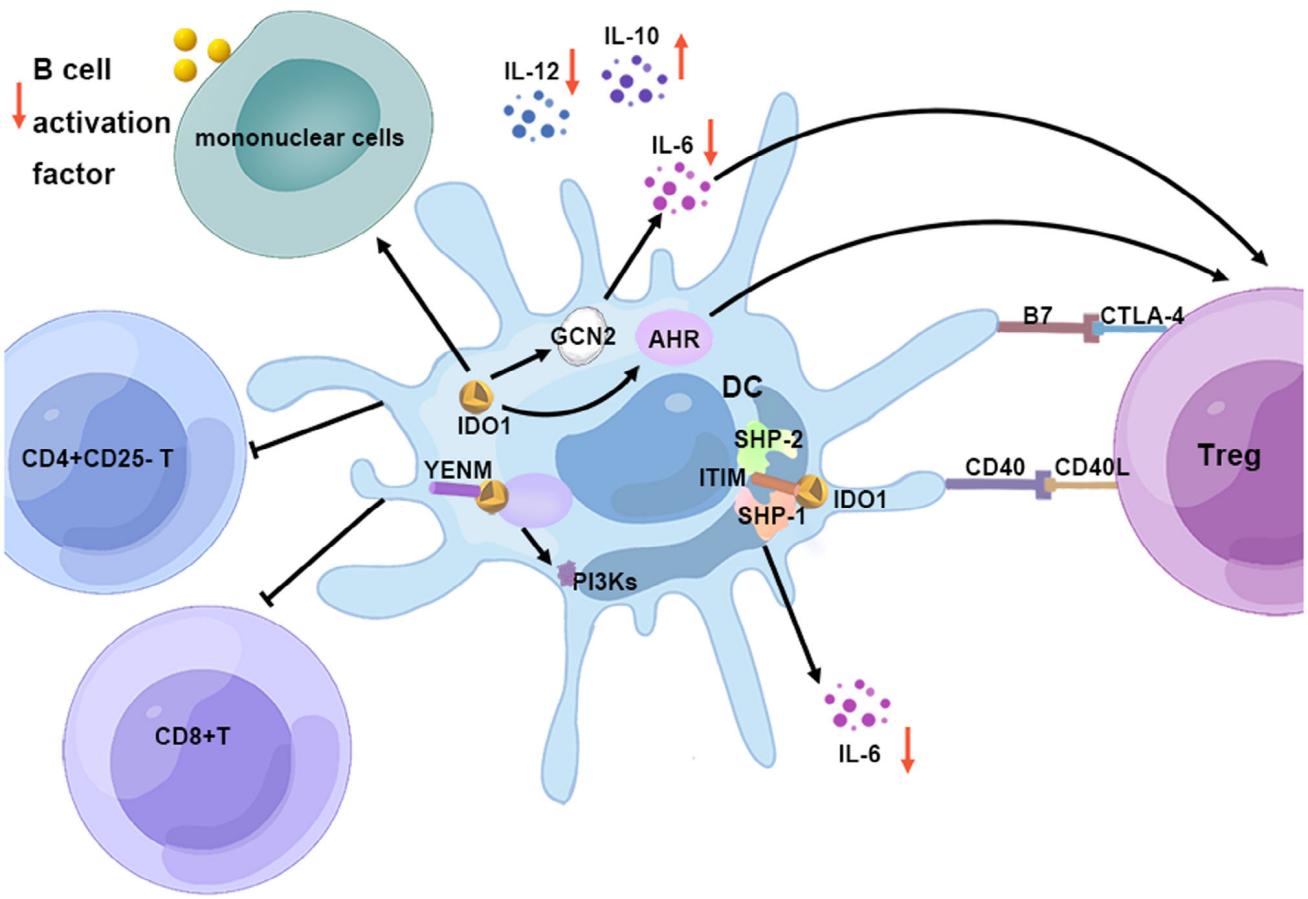
Mechanism of IDO^+^ DC‐mediated immunosuppression. IDO^+^ DCs modulate the immune landscape by influencing monocytes to secrete B cell activation factors, curbing the proliferation of CD4^+^CD25^−^ T cells, and inducing apoptosis in CD8^+^ T cells. They also suppress the release of IL‐6 and IL‐12 while promoting IL‐10 secretion, thus affecting the antigen‐presenting capacity of DCs and orchestrating immune tolerance through a blend of metabolic and nonenzymatic functions.

IDO's enzymatic function is complemented by its nonenzymatic roles. TGF‐β may activate IDO1 in DCs to mediate nonenzymatic signals through the phosphorylation and activation of the immune receptor Tyr‐based inhibitory motif (ITIM). The ITIM on IDO serves as a docking site for Tyr phosphatases SHP‐1 and SHP‐2, initiating the noncanonical NF‐κB pathway. This pathway inhibits IL‐1 receptor‐associated kinase 1, averting the production of the proinflammatory cytokine IL‐6. Furthermore, IDO1 harbors a YENM motif that can directly interact with p85, activating the class IA PI3Ks p110 subunit, which anchors IDO1 to the early endosome and subsequently activates the immune regulation pathway in pDCs. The results emphasize the crucial role of IDO1 in the long‐term regulation of immune responses within DCs (Figure 3).^189^

#### 4.2.2 Laccase domain‐containing 1

LACC1, also recognized as C13orf31 or FAMIN, is a zinc‐dependent endogenous isocyanic acid synthase enzyme,^190^ belonging to the domain of unknown function 152 family.^191^ Studies have linked LACC1 to various conditions, including juvenile idiopathic arthritis,^192^ cognitive disorder,^193^ and Still's disease.^194^ This enzyme enhances lipid synthesis de novo and concurrently stimulates FAO and glycolysis, leading to a boost in ATP production.^191^ LACC1 exhibits a quartet of enzymatic activities: adenosine deaminase, purine nucleoside phosphorylase, methylthioadenosine phosphorylase, and adenosine phosphorylase, participating in the synthesis and degradation of purine derivatives such as adenosine, hypoxanthine, guanine, and adenine. Predominantly expressed in DCs and macrophages, elevated LACC1 in DCs can modulate the response to class I and class II antigens, slowing antigen uptake, processing, and presentation. It regulates DC metabolism to mediate immune tolerance and dampen T cell responses triggered by viruses. The metabolite inosine, released by DCs, can inhibit the activation of naïve CD4^+^ and CD8^+^ T cells, further intensifying the inhibitory signal and reducing the antitumor immune response.^194^

### 4.3 Immunometabolic checkpoints on Macrophage

#### 4.3.1 Glyceraldehyde‐3‐phosphate dehydrogenase

GAPDH, a 335‐amino‐acid oxidoreductase, is central to glycolysis. It catalyzes the conversion of 3‐phosphoglyceraldehyde to 1,3‐diphosphoglycerate, generating NADH and participating in ATP production via the mitochondrial electron transport chain.^195^ ^, .^ ^196^ Highly conserved across species and broadly expressed, GAPDH serves as a reference gene or static marker in numerous gene and protein expression studies.^197^, ^198^ Beyond its metabolic role, GAPDH exhibits moonlighting characteristics, binding to nucleic acids to regulate transcription and interacting with neurodegenerative disease‐related proteins to promote neuronal apoptosis.^197^, ^199^, ^200^ In addition, there is also evidence that GAPDH has an immune regulation function. GAPDH can strongly inhibit the adhesion, diffusion, and phagocytosis of macrophages stimulated by LPS without affecting cell viability. By binding to mRNA transcripts containing AU‐rich elements, GAPDH regulates and reduces the production of tumor TNF‐α, while inducing the production of IL‐10 in macrophages stimulated by LPS in a dose‐dependent manner.^195^, ^201^ Moreover, GAPDH and its driven NADH can induce a distinct macrophage phenotype characterized by M1 and M2 markers while promoting macrophage differentiation into the anti‐inflammatory M2 phenotype.^195^

#### 4.3.2 Aconitate decarboxylase 1

ACOD1, also known as immune‐responsive gene 1 protein (IRG1), is a homodimeric enzyme primarily localized in macrophage mitochondria and is essential for macrophage metabolic reprogramming.^202^ LPS, cytokines IFN‐γ, TNF, and TLR agonists robustly upregulated the expression of ACOD1 in macrophages via TLR signaling. Furthermore, there was a significant synergistic effect between IFN‐γ and TNF on the induction of ACOD1. Additionally, low‐level expression of ACOD1 was also induced by IFN‐β and IL‐1β. Remarkably increased expression of ACOD1 was observed in models of Listeria monocytogenes and Toxoplasma gondii infection.^202^ In activated macrophages, ACOD1 catalyzes the decarboxylation of cis‐aconitic acid, an intermediate in the TCA cycle, to generate inhibitory itaconate. The accumulation of itaconate can impact the TCA cycle and cellular energy metabolism, modulate the activities of aldolase A and GAPDH, suppress glycolysis, regulate the expression of inflammatory genes, restrict the response of type I IFN, inhibit the production of TLR‐triggered proinflammatory cytokines TNF‐α, IL‐6 and IFN‐β in LPS‐tolerant macrophages, and impede the activation of IRF3.^203^, ^204^, ^205^, ^206^ The primary function of itaconate in most cases is to establish immune tolerance and regulate inflammatory progression by primarily inhibiting immune responses.^207^ In a study on influenza A virus infection, researchers observed an increase in the level of ACOD1 in the lungs of mice with higher itaconate concentration. Itaconate inhibits the IFN response in infected cells by suppressing STAT1 phosphorylation without affecting viral RNA replication, thereby limiting lung inflammation and disease severity.^208^ It was also discovered that itaconate is capable of inhibiting NLRP3 activation by modifying the specific cysteine (C548) on the inflammasome NLRP3 and disrupting the interaction between NLRP3 and NEK7, thereby exerting immunomodulatory effects to prevent excessive inflammation.^209^ The activation of Nrf2 signal transduction by itaconate occurs through the alkylation of KEAP1 cysteine residues, leading to the inhibition of inflammation and oxidative stress in macrophages and tissues.^205^, ^210^ Itaconate can also bind to TET DNA demethylase, inhibit α‐ketoglutaric acid, down‐regulate NF‐κB and STAT target genes, and thus inhibit inflammatory responses.^211^ In peritoneal tumors, OxPhos of TAM is increased, and itaconate regulates mitochondrial ROS production in TAM and further regulates MAPK activation in tumor cells. Itaconate is a key metabolic component of the crosstalk between tumor and TAM, and TAM can promote tumor progression through ACOD1–itaconate.^212^ Activated CD8^+^ T cells are unable to endogenously secrete itaconate, but can uptake exogenous itaconate released by other cells. which can reduce the level of phospho‐STAT3 in the JAK‐STAT signaling pathway, inhibit the biosynthesis of aspartic acid and serine/glycine, inhibit the activation of CD8^+^ T cells, and inhibit the proliferation of activated CD8^+^ T cells. In addition, itaconate can also inhibit the specific killing of CD8^+^ T cells by inhibiting the secretion of cytokines IL2, TNFα, and IFN‐γ, and reducing the levels of granzyme B and perforin.^213^

However, some studies have also demonstrated that ACOD1 can aid in eradicating bacteria in certain bacterial infections and maintaining the body's homeostasis. In the investigation of Clostridium difficile infection, it was observed that Clostridium difficile infection strongly induces the expression of ACOD1, and the itaconate derived from ACOD1 can directly hinder bacterial replication by altering host cell metabolism and mediating macrophage defense against Candida difficile.^214^ Transcription factor EB (TFEB) serves as a key regulator of phagocytosis‐lysosomal‐mitochondrial crosstalk in macrophages, leading to the activation of lysosomal and metabolism‐related gene expression. Upon bacterial stimulation, TFEB is activated and directly promotes ACOD1 transcription, resulting in itaconate production. Subsequently, itaconate is transported to the vacuole containing Salmonella through the Irg1–Rab32–BLOC3 system, selectively inhibiting bacterial proliferation to safeguard the phago‐lysosomal compartment from being overtaken by bacterial growth.^215^ Studies on bacterial infections in the respiratory tract have demonstrated that secretory itaconate, acting as a paracrine signaling molecule, is capable of activating oxoglutarate receptor 1 to facilitate the upregulation of airway barrier function and the promotion of mucociliary clearance by innate immune cells. This process serves to timely remove inhaled pathogens and protect the body.^211^ Further research has demonstrated that ACOD1 suppresses the growth of bacteria in a manner dependent on isocitrate lyase (ICL). ICL is one of the key enzymes of Mycobacterium tuberculosis (Mtb) glutaric acid shunting, which can provide carbon for Mtb to carry out gluconeogenesis, maintain Mtb survival and virulence. Itaconate is an inhibitor of ICL, which can effectively inhibit the growth of Mtb and control the progression of the disease.^216^, ^217^

In addition to macrophages, ACOD1 can also act on other immune cells. The metabolite of ACOD1, itaconate, can also reduce the secretion of TNF‐α and IL‐6 by DC, increase the secretion of IL‐10, and induce the expression of PD‐L1 in the stimulator of IFN genes‐IRF3/IRF7 dependent pathway, restrict the activation of CD8^+^ T cells, reduce the reactivity of CD8^+^ T cells, and thus affect the immune state of the body.^218^ Tumor‐secreted GM‐CSF induces ACOD1 expression in neutrophils through the STAT5‐C/EBPβ axis. In tumor‐infiltrating neutrophils (TINs), ACOD1 is the most significantly upregulated metabolic enzyme. Activation of ACOD1 reduces TIN ferroptosis by generating itaconate and activating Nrf2‐mediated antioxidant response, thereby sustaining the abundance of TINs in tumor metastasis. However, following ACOD1 knockout, there is a decrease in the number of surviving TINs, reduced accumulation of TINs, and enhanced adaptive immunity.^219^

#### 4.3.3 Laccase domain‐containing 1

LACC1 in mouse bone marrow‐derived macrophages is activated by LPS or poly‐I:C, and can convert l‐citrulline into an RCS‐like cytotoxin HNCO and l‐ornithine.^194^ l‐Ornithine is then decarboxylated by ODC1 to produce putrescine, a precursor to polyamines, which are crucial for cell proliferation, differentiation, and disease progression.^220^ LACC1 protects macrophages from pathogen‐induced apoptosis by inhibiting the production of proinflammatory cytokines through the polyamine pathway.^190^

#### 4.3.4 IL‐4 induced 1

IL4I1 expression increases during macrophage differentiation and can be induced by various cytokines and TLR agonists. IL4I1 promotes M2 macrophage polarization and reduces M1 polarization, inhibiting the proinflammatory state of macrophages. It also suppresses T cell activation by consuming l‐tryptophan and l‐arginine, leading to IL‐10 production.^221^

### 4.4 Immunometabolic checkpoints on NK cells

Beyond its influence on T cells, CD38 is a pivotal regulator of immune function, particularly modulating the activity of NK cells. Constitutive expression of CD38 in NK cells is crucial, as the interaction between CD38 and CD16 equips CD16‐expressing NK cells with an effector cytotoxicity phenotype. This interaction bolsters NK cell activation and the release of cytokines, such as granzyme and IFN‐γ.^222^, ^223^ Elevated CD38 expression is observed in rheumatoid arthritis (RA), where higher levels of CD38^+^ cells correlate with the presence of rheumatoid factors. CD38's regulatory role extends to dampening TNF‐α levels and enhancing IFN‐γ secretion in NK cells by suppressing Sirtuin 6 expression, inhibiting the differentiation of monocytes into Treg cells, leading to immune imbalance. CD38^+^ NK cells also activate mTOR signaling in cocultured CD4^+^ T cells, significantly altering the Th1/Th2 and Th17/Treg ratios and fostering inflammation.^224^

However, the role of CD38 in NK cells varies among different subsets. The expression level of CD38 in NK cells can be increased by IL‐12, IL‐15, and IL‐18.^225^ In secondary lymphoid organs, the CD16^−^CD56^bright^ NK cell subset, which is less mature, releases anti‐inflammatory cytokines upon stimulation and suppresses CD4^+^ T cell proliferation through a G protein‐coupled receptor and CD38‐dependent pathway.^226^ In HIV patients, the proportion of CD38^+^CD39^+^ NK cells is closely tied to disease progression, with these cells expressing high levels of inhibitory receptors LAG‐3 and TIM‐3, leading to increased TGF‐β and IL‐10 expression and a regulatory phenotype.^225^ In systemic lupus erythematosus patients, overexpression of CD38 in NK cells impairs the response of surface receptors SLAMF1 and SLAMF7 to stimulation, compromising NK cell function and contributing to disease pathogenesis.^227^

## 5 THERAPEUTIC TARGETING OF IMMUNOMETABOLISM

**FIGURE 4:**
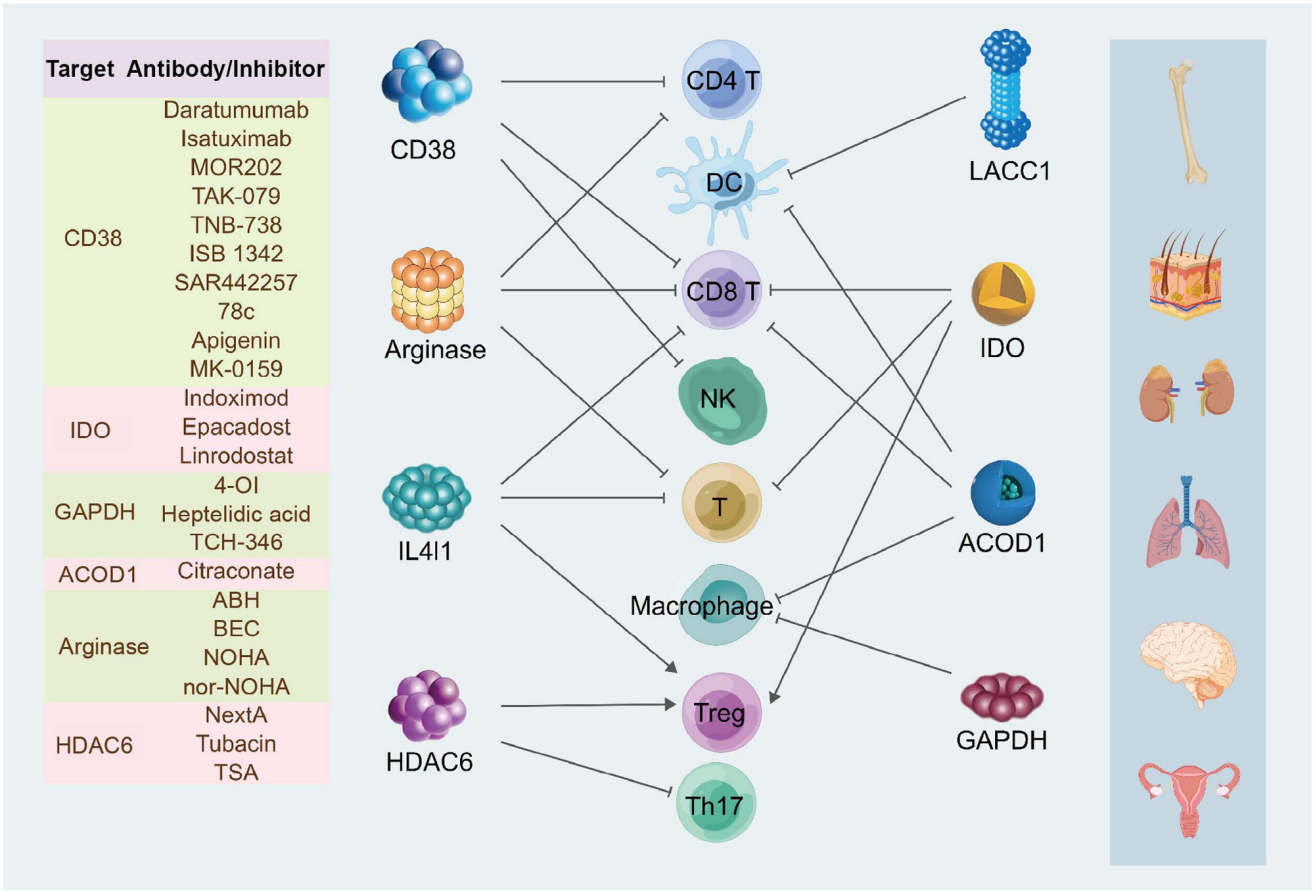
Antibodies and inhibitors of immune metabolic enzymes. Inhibitors and antibodies targeting metabolic enzymes with immunomodulatory functions have shown promising potential for treating various cancers.

Drawing upon the structural and metabolic profiles of these enzymes, the scientific community has engineered an array of inhibitors and antibodies specifically designed to target their unique attributes. In addition, CAR T cell therapies have been innovated, leveraging enzyme targets to bolster their therapeutic efficacy (Figure 4).

### 5.1 Targeting CD38

The landscape of CD38‐targeted therapeutics has seen remarkable progress, with the development of anti‐CD38 antibodies, small molecule inhibitors, and CD38‐directed CAR‐T/CAR‐NK therapies taking center stage.

Biologically inspired therapies, such as anti‐CD38 monoclonal antibodies (mAbs), have shown considerable promise in clinical trials. A plethora of CD38 antibodies is under global development, with daratumumab and isatuximab leading the way, having received market authorization. Both mAbs are approved for treating MM.^228^ Daratumumab, a human IgG1κ mAb, targets CD38's two β chains with high specificity for malignant tumor cells. Its antitumor effects are multifaceted, encompassing antibody‐dependent cytotoxicity (ADCC), complement‐dependent cytotoxicity (CDC), macrophage activation via FcγR for antibody‐dependent cell phagocytosis (ADCP), direct induction of apoptosis, and reduction of immunosuppressive adenosine secretion. Daratumumab also depletes MDSCs, Tregs, Bregs, and other immune subsets, while promoting CD4^+^ and CD8^+^ T cell expansion.^229^, ^230^, ^231^, ^232^, ^233^, ^234^ Daratumumab is approved for MM treatment as a monotherapy or combined with melphalan, immunomodulatory drugs, and proteasome inhibitors.^228^ In phase I/II GEN501 and phase 2 SIRIUS trials for relapsed or refractory multiple myeloma (RRMM), daratumumab (16 mg/kg) achieved overall response rates (ORRs) of 36 and 29%, respectively. The combined clinical benefit rate (ORR+MR) was 37.2%, with 83.1% of patients attaining stable disease (SD) or better, showcasing its efficacy.^235^ The phase III clinical trial of carfilzomib, dexamethasone, and daratumumab for RRMM revealed significantly improved median progression‐free survival (PFS) and median overall survival (mOS) for the carfilzomib, daratumumab, and dexamethasone (KdD) group.^236^, ^237^

Although daratumumab has shown encouraging results in the treatment of MM, it has been found in studies that some patients fail to benefit from it, and eventually, all patients face the dilemma of disease progression. In addition to inherent resistance to daratumumab due to interindividual variability in CD38 expression levels, acquired resistance can also develop in patients receiving this treatment. Studies have demonstrated that daratumumab treatment leads to the depletion of CD38 cells. However, given the expression of CD38 in MM cells and its widespread distribution in NK, T, and B cells, such depletion will inevitably impact the immune status of the organism. Furthermore, the CD38–dara complex formed by CD38 and daratumumab is phagocytosed by monocytes and granulocytes, leading to MM cells displaying altered surface antigen profiles and thus promoting immune evasion. Additionally, daratumumab induces self‐destruction of CD38‐expressing NK cells through ADCC, and depletion of NK cells consequently impacts the NK‐mediated ADCC activity of daratumumab. MM cells also exhibit overexpression of CD47, which binds to signal‐regulated protein α on TAM, leading to the inhibition of ADCP and promotion of immune escape in tumor cells. Additionally, homeostasis mechanisms such as humoral regulators or complement inhibitors hinder daratumumab‐mediated CDC, thereby facilitating tumor immune evasion and compromising the efficacy of daratumumab treatment.^234^, ^238^ Ongoing research is being conducted to explore new combination therapies to further broaden the scope of daratumumab, with multiple studies initiating large‐scale phase III clinical trials (Table 1).

**TABLE 1:** Ongoing phase III clinical trial of anti‐CD38 mAbs.

Anti‐CD38 mAbs	Strategy	NCT number	Disease	Enrollment patients
Daratumumab	IberDd versus DVd	NCT04975997	MM	864
	BCD‐264 combined with daratumumab	NCT06296121	MM	252
	D versus DPd versus DRd versus DKd versus Pd versus Rd versus Kd	NCT05438043	MM	500
	TDPd versus DPd versus TDd	NCT05455320	RRMM	810
	ED versus E versus DPd	NCT05020236	MM	762
	tDR versus DRT versus DRd	NCT05552222	MM	1590
	Dd versus DR	NCT03937635	Smoldering plasma cell myeloma	288
	EDR versus DRd	NCT05623020	MM	966
	No Intervention versus R versus RD	NCT04071457	MM	1100
	DVRd versus DVRdKcF	NCT05257083	MM	750
	Daratumumab versus placebo	NCT05403138	Neuromyelitis optica, NMO spectrum disorder	72
	DdR versus VDdR	NCT04566328	Plasma cell myeloma, RISS stage I/II plasma cell myeloma	1450
	VdR versus DdR	NCT05561387	Plasma cell myeloma	510
	KdD	NCT04513639	MM	176
	Allogeneic stem cell transplantation versus KRd or erd or DVd or DRd or IRd or PVd or KDd	NCT05675319	MM	482
Isatuximab	The administration of SRVd occurs via various routes	NCT05804032	MM	514
	The administration of Sdp occurs via various routes	NCT05405166	Plasma cell myeloma recurrent	534
	SRVd + autologous stem cell transplant versus SRVd	NCT05665140	MM	100

All the data in the above table are from ClinicalTrials.gov.

Abbreviations: C, cilta‐cel; c, cyclophosphamide; D, daratumumab; d, dexamethasone; e, elotuzumab; E, elranatamab; F, fludarabine; I, ixazomib; Iber, iberdomide; K, carfilzomib; MM, multiple myeloma; P, pomalidomide; p, pomalyst; R, lenalidomide; RRMM, relapsed or refractory multiple myeloma; S, isatuximab; T, talquetamab; t, teclistamab; V, bortezomib.

Isatuximab, an IgG‐κ chimeric anti‐CD38 mAb,^232^ isatuximab can induce apoptosis through both Fc‐dependent and Fc‐independent activities by inhibiting CD38 enzyme activity. The Fc‐dependent mechanisms include CDC, ADCC, and ADCP, while the Fc‐independent mechanisms involve caspase‐dependent and lysosome‐associated pathways. The fragment of daratumumab that binds to antigens and its Fc fragments are sufficient to activate NK cells, whereas the activation of NK cells by isatuximab depends on the cross‐linking of CD16 and CD38 with full‐length isatuximab.^239^, ^240^ Isatuximab can inhibit Treg proliferation and migration, suppress IL‐10 secretion, and enhance the cytotoxic capabilities of CD8^+^ T cells and NK cells against tumor cells.^241^ Isatuximab's combination with immunomodulators shows synergistic potential in preclinical studies.^242^ Isatuximab can bind to CD38 molecules previously bound by HB‐7 or AT13/5 antibodies, making it primarily suitable for adult patients with RRMM who have received prior treatment. It can be administered in combination with dexamethasone, pomalidomide, or carfilzomib.^228^ In phase 3 trials, this combination demonstrated significant improvements in PFS, with promising results anticipated in upcoming trials.^242^, ^243^ The ongoing phase 3 trials of isatuximab are anticipated to yield positive results soon, and we eagerly await the outcome (Table 1).

In addition to the approved CD38 antibodies, a variety of other CD38‐targeting antibodies are currently in clinical trials, showcasing the diversity of therapeutic strategies under investigation. MOR202 is a fully human CD38 antibody that primarily induces tumor cell lysis through ADCC and ADCP mechanisms, demonstrating promising therapeutic efficacy in clinical trials for RRMM at stage I/IIa.^244^ Lenalidomide has been found to potentially enhance the macrophage‐mediated cytotoxicity dependent on MOR202 via the vitamin D pathway.^245^ TAK‐079 is an all‐human IgG1λ mAb that specifically targets CD38, inhibits its enzymatic activity, and induces apoptosis and cytolysis through ADCC and CDC mechanisms.^246^ In arthritis studies, TAK‐079 has shown potential for prophylactic administration, inhibiting the progression of arthritis and joint damage by reducing various immune cells, including lymphocytes, NK cells, B cells, and T cells.^247^ TNB‐738 is a human anti‐CD38 biparatopic antibody with high affinity for two distinct epitopes on CD38, synergistically achieving maximal CD38 inhibition without inducing cytokine production in CD38^+^ T cells.^248^ ISB 1342 is a bispecific antibody targeting both CD38 and CD3, potentially allowing for combination or sequential use with daratumumab.^249^ SAR442257 is a trispecific T cell‐engaging antibody that targets CD38 on MM cells, CD3 on T cells, and CD28 on both cell types, offering extensive potential for treating patients resistant to both CD38 monoclonal antibodies and BCMA‐targeted therapy.^250^

The expression of CD38 has been linked to increased PD‐1 expression, contributing to resistance against PD‐1/PD‐L1 blockade in some studies. Tumor cells may inhibit ADCC mediated by anti‐CD38 monoclonal antibodies through the PD‐L1/PD‐1 pathway, and the subsequent upregulation of CD38 expression can inhibit CD8 T cells. Studies combining CD38 antibodies with PD‐L1 monoclonal antibodies have shown promise in preclinical models,^87^, ^96^, ^240^ but clinical trials combining durvalumab with daratumumab and atezolizumab with isatuximab have not met expectations,^251^, ^252^ indicating a need for further investigation.

Small molecule inhibitors targeting CD38 have emerged as a novel class of drugs with significant potential. Thiazoloquin(az)olin(on)es, such as compound 78c, are highly compatible with CD38 and exhibit excellent cell permeability, high steady‐state distribution volume, and long half‐life.^253^ Compound 78c is a potent reversible inhibitor of CD38, specifically suppressing its NADase activity without affecting other isoenzymes or homologous enzymes.^254^ In vivo, 78c elevates NAD levels, potentially improving age‐related conditions and extending the lifespan and health span of mice without significant antitumor effects, suggesting its primary therapeutic effect is through metabolic regulation.^254^, ^255^, ^256^

Apigenin (4′,5,7‐trihydroxyflavone), a natural flavonoid, has shown promise in decreasing CD38 expression and inhibiting its activity, with potential applications in a variety of diseases, including cancer and central nervous system inflammation.^88^, ^255^, ^257^, ^258^, ^259^

MK‐0159 is another compound that effectively inhibits CD38 enzymatic activity and restores NAD and NADP levels in cells, presenting a promising avenue for CD38 inhibitor research.^260^

In addition to antibodies and small molecule inhibitors targeting CD38, numerous studies have focused on developing anti‐CD38 CAR cells, including the engineering of anti‐CD38 CAR‐T cells and CAR‐NK cells through genetic technology. These can be utilized for effective tumor treatment by transfusing specific T or NK cells capable of recognizing and attacking CD38^+^ tumor cells. To prevent the autolysis reaction caused by the binding of anti‐CD38 CAR‐T to CD38 on the cell surface, some researchers have attempted to combine CD38 antibody with anti‐CD38 CAR‐T, resulting in significant and specific tumor cell killing in both in vitro and mouse experiments.^102^ CD38 CAR‐T cells have been developed for phase 1/2 clinical trials, and a double‐targeted DCAR‐T cell, which targets CD38 and SLAMF7, has also been constructed for the treatment of MM. This approach can rapidly induce an immune response without causing toxicity.^261^ CD38‐LMP1 tandem CAR‐T cells were engineered for the treatment of NK/T cell lymphoma (NKTCL). Tandem CAR‐T cells exhibited superior cytolytic activity against tumor cells compared to single‐target CAR‐T cells in both in vitro and in vivo experiments. Furthermore, even if one target molecule was lost during treatment, the tandem CAR‐T cells still demonstrated synergistic enhancement by recognizing the target and exerting cytolytic activity against NKTCL cells.^262^ Furthermore, the dual split CAR strategy is employed to simultaneously target CD38 and CD138 for the construction of CAR‐T cells. Interestingly, this dual split CAR‐T cell therapy may exhibit potential therapeutic benefits for patients who are refractory to daratumumab.^263^ The CD38‐targeted CAR was engineered utilizing the NK cell line NK92. The CAR‐NK cells demonstrated enhanced expression of IFN‐γ, perforin, and granzyme, and exhibited CD38‐dependent lysis of Daudi and Jurkat cells.^264^ Another form of CAR‐NK involves the use of CRISPR–Cas9 technology to knock out CD38, followed by mRNA electroporation to introduce an enhanced CAR targeting CD38. This CD38‐knockout NK cell with anti‐CD38 CAR can evade NK cell‐mediated autophagy and enhance cytotoxic activity against CD38^−^ expressing MM cells.^265^

### 5.2 Targeting IDO/TDO

IDO, an immune checkpoint enzyme with well‐defined biochemical properties, is an attractive target for small‐molecule inhibitors. Currently, 11 IDO inhibitors are in clinical investigation,^266^ including two in phase III and two in phase II trials. IDO inhibitors as monotherapy have generally shown limited efficacy in tumor treatment, and research has indicated a complex interplay between IDO1 expression, PD‐1 expression, and immune therapy resistance.^267^, ^268^, ^269^, ^270^ The combination of IDO inhibitors with PD‐1/PD‐L1 monoclonal antibodies is a strategy being explored to modulate the tumor immunosuppressive microenvironment and enhance the efficacy of cancer therapy. Clinical trials of IDO inhibitors have yielded mixed results, and ongoing trials are being closely monitored for further insights (Tables 2 and 3).

**TABLE 2:** Clinical trials of IDO inhibitors with published results.

IDO inhibitor	Strategy	Phase	Tumor type	Enrollment patients	Results
Indoximod	Combined with adenovirus‐p53 transduced DC vaccine	I/II (NCT01042535)	Male breast cancer, recurrent breast cancer, etc.	44	Among the 21 participants involved in the study analysis, four were SD after 16 weeks.
	Combined with sipuleucel‐T	II (NCT01560923)	Metastatic prostate cancer	47	The immune response of patients’ PBMC to immune protein PA2024 was determined by ELISPOT assay: sipuleucel‐T + placebo: 29.6 spots per mL; sipuleucel‐T + indoximod 25.45 spots per mL.
	Combined with docetaxel or paclitaxel	II (NCT01792050)	Metastatic breast cancer	169	The median PFS was 6.8 months in the indoximod group and 9.5 months in the placebo group.^271^
	Combined with gemcitabine, Nab‐paclitaxel	I/II (NCT02077881)	Metastatic pancreatic cancer	104	ORR: 46.2%, mOS: 10.9 months, median PFS: 5.8 months.^272^
Linrodostat (BMS‐986205)	Combined with nivolumab	III (NCT03329846)	Melanoma skin cancer	20	The drug‐related SAEs, any cause AEs and drug‐related AEs in the nivolumab + BMS‐986205 group were higher than those in the nivolumab group.
	Combined with nivolumab, ipilimumab	I/II (NCT02658890)	Advanced cancer, melanoma, NSCLC	630	According to the treatment of patients with different tumor types at different doses of BMS‐986205, its ORR ranges from 0 to 42.9%.
Epacadostat (INCB024360)	Combined with pembrolizumab	III (NCT03361865)	Urothelial cancer	93	Epacadostat + pembrolizumab group ORR: 31.8% (14/44), placebo + pembrolizumab group ORR: 24.5% (12 out of 49).
	Combined with pembrolizumab	III (NCT03374488)	Urothelial cancer	84	Epacadostat + pembrolizumab group ORR: 21.4% (9/42), placebo + pembrolizumab group ORR: 9.5% (four out of 42).
	Combined with pembrolizumab	III (NCT03260894)	Renal cell carcinoma	129	Pembrolizumab + epacadostat group CR+ PR: 31.3% (20 out of 64), sunitinib or pazopanib group CR + PR: 29.2% (19 out of 65).
	Combined with pembrolizumab	III (NCT03358472)	Head and neck cancer	89	Epacadostat + pembrolizumab group ORR: 31.4% (11 out of 35) pembrolizumab group ORR: 21.1% (four out of 19), extreme group ORR: 34.3(12 out of 35).
	Combined with MELITAC 12.1 peptide vaccine	II (NCT01961115)	Melanoma	11	For seven patients with advanced melanoma who did not receive anti‐PD‐1 and anti‐PD‐L1 therapy before, IHC staining showed that the amount of CD8^+^ cells was four times as before treatment after the end of combination therapy.
	Combined with pembrolizumab and chemotherapy	I/II (NCT03085914)	Solid tumor	70	According to different treatment strategies, the ORR range after 18 weeks of treatment is 12.5–55.6%.
	Combined with durvalumab	I/II (NCT02318277)	Solid tumor	176	During phase II, epacadostat (100 mg) + Durvalumab (10 mg/kg) group ORR: 12.2% (six out of 49), epacadostat (300 mg) + durvalumab (10 mg/kg) group ORR: 12.9% (12 out of 93).
	Combined with SD‐101 and radiotherapy	I/II (NCT03322384)	Advanced solid tumors, lymphoma	20	ARR: 28.6% (four out of 14)
	Monotherapy	II (NCT01822691)	Myelodysplastic syndromes	15	SD: 80% (12 out of 15)
	Combined with MK‐3475	I/II (NCT02178722)	Microsatellite‐instability high colorectal cancer, endometrial cancer, head and neck cancer, etc.	444	Except for the lowest dose group, SAE occurred in 40% or more of the other dose groups in the phase I experiment. In the phase II trial, ORR was 60.5% (26 out of 43) in patients with melanoma‐immune checkpoint‐naïve and 43.8% (seven out of 16) in patients with microsatellite‐instability high colorectal cancer.
	Combined with pembrolizumab	II (NCT03322540)	Lung cancer	154	Epacadostat + pembrolizumab group ORR: 32.5% (25 out of 77), pembrolizumab + placebo group ORR: 39% (30 out of 77)
	Combined with pembrolizumab and platinum‐based chemotherapy	II (NCT03322566)	Lung cancer	233	Pembrolizumab + chemotherapy + epacadostat group ORR: 26.4% (24 out of 91), pembrolizumab + chemotherapy + placebo group ORR: 44.8% (39 out of 87)
	Combined with pembrolizumab	III (NCT02752074)	Melanoma	706	Pembrolizumab + epacadostat group median PFS: 4.7 months, pembrolizumab + placebo group median PFS: 4.9 months
	Combined with CDX‐1401, poly‐ICLC	I/II (NCT02166905)	Fallopian tube carcinoma, etc.	40	Without IDO1 inhibitor INCB024360 group PFS rates: 0.78, with IDO1 inhibitor INCB024360 group PFS rates: 0.54, exploratory cohort PFS rates: 0.5
	Combined with nivolumab	I/II (NCT02327078)	B‐cell malignancies, colorectal cancer, etc.	307	In the phase II experiment, patients with various tumor types were treated with different doses of epacadostat, resulting in significant variations in treatment response, with an ORR range from 0% to 62%. The OS rate of glioblastoma patients was 0.458 (*n* = 33).

All the data in the above table are from ClinicalTrials.gov except for those whose remarks are quoted from the literature.

Abbreviations: SD, stable disease; PFS, progression‐free survival; SAEs, serious adverse events; AEs, adverse events; ORR, objective response rate; CR, complete response; PR, partial response; ARR, abscopal response rate; mOS, median overall survival; SD‐101, a Toll‐like receptor 9 agonist that can stimulate human plasma cells like DC to release IFN‐α and mature into highly effective antigen‐presenting cells, enhancing innate and adaptive immune responses; CDX‐1401, DEC‐205/NY‐ESO‐1 fusion protein; poly‐ICLC, a TLR‐3/MDA‐5 agonist; MK‐3475, pembrolizumab.

**TABLE 3:** Clinical trials of IDO inhibitors without published results.

IDO inhibitor	Strategy	Phase	Tumor type	Enrollment patients
Indoximod	Combined with ipilimumab, pembrolizumab, nivolumab	II (NCT02073123)	Melanoma	132
	Combined with temozolomide	I/II (NCT02052648)	Malignant brain tumor	160
	Combined with chemotherapy, radiotherapy	II (NCT04049669)	Glioblastoma, medulloblastoma, etc.	Estimated enroll 140
Linrodostat (BMS‐986205)	Combined with nivolumab	III (NCT03661320)	Urinary bladder neoplasms muscle‐invasive bladder cancer	861
	Combined with nivolumab	II (NCT04106414)	Endometrial adenocarcinoma, endometrial carcinosarcoma	24
	Combined with relatlimab, nivolumab	I/II (NCT03459222)	Advanced cancer	Estimated enroll 255
	Combined with nivolumab	II (NCT03854032)	Lip oral cavity squamous cell carcinoma, pharynx larynx squamous cell carcinoma	45
Epacadostat (INCB024360)	Combined with pembrolizumab	II (NCT03414229)	Sarcoma	30
	Combined with DPX‐survivac, cyclophosphamide	I/II (NCT02785250)	Recurrent epithelial ovarian cancer, recurrent fallopian tube cancer, etc.	85
	Combined with retifanlimab	II (NCT04463771)	Endometrial cancer	260
	Combined with FPV‐Brachyury, MVA‐BN‐Brachyury, M7824, N‐803	I/II (NCT03493945)	Prostate cancer, solid tumor	Estimated enroll 113
	Combined with bevacizumab	II (NCT03532295)	Glioma	Estimated enroll 55

All the data in the above table are from ClinicalTrials.gov except for those whose remarks are quoted from the literature.

Abbreviations: AEs, adverse events; ARR, abscopal response rate; CR, complete response; DPX‐Survivac, it consists of five unique HLA‐restricted peptides (HLA‐A1, A2, A3, A24, B7) derived from the survivin protein and adjuvants encapsulated in nano‐lipid particles; M7824, a bifunctional fusion protein fused with IgG1 mAb targeting PD‐L1 protein and human TGF‐β receptor type II; mOS, median overall survival; N‐803, IL15RaFc superagonist; ORR, objective response rate; PFS, progression‐free survival; PR, partial response; SAEs, serious adverse events; SD, stable disease.

#### 5.2.1 Inhibitors indoximod and epacadostat to IDO/TDO

Indoximod is a Trp analogue that can be recognized by mTORC1 as Trp and directly acts on immune cells to reverse the IDO/TDO pathway‐mediated immunosuppression.^175^, ^273^, ^274^ Its cellular mechanism of action is to relieve the inhibition of Teff cells in tumors, limit the generation of Tregs, and reprogram Tregs into Th17 helper cells in draining lymph nodes.^274^ The result of a phase II single‐arm clinical trial on pembrolizumab combined with indoximod in the treatment of advanced melanoma showed that the median PFS of 89 patients in the nonocular melanoma cohort treated with combination therapy was 12.4 months. The objective response rate was 51%, the complete response rate was 20%, and the disease control rate was 70%. The combination therapy was well‐tolerated, with side effects similar to those of pembrolizumab alone. Although this single‐arm study cannot conclude that the use of indoximod in combination with pembrolizumab is significantly better than using pembrolizumab alone, such results are encouraging.^175^

Epacadostat serves as a specific enzyme inhibitor of IDO1, precisely suppressing its activity by forming a complex with the heme group of IDO1.^175^, ^275^ This interaction is facilitated through the oxygen atom of the hydroxyamidine group.^276^ Preclinical studies indicate that epacadostat selectively inhibits the Trp‐degrading activity of human IDO1, with minimal impact on IDO2 and TDO2. In cocultures with human allogeneic lymphocytes and DCs or tumor cells, epacadostat promotes the proliferation of effector T cells and NK cells, reduces the maturation of Tregs from immature T cells, and increases the number of CD86^+^ DCs.^277^ However, a phase III clinical trial for patients with unresectable stage III or IV melanoma found no significant difference in PFS, overall survival, or severe adverse events between the epacadostat and pembrolizumab combination therapy and the placebo plus pembrolizumab group.^278^ This suggests that the addition of epacadostat to pembrolizumab does not enhance the therapeutic outcome of pembrolizumab monotherapy.

The potential of IDO1 inhibitors to augment the anticancer effects of PD‐1 antibodies is still under investigation. Despite the uncertainty, research continues to explore the use of epacadostat in cancer therapy. Discrepancies between phase I/II and phase III trials have been attributed to differences in the patient populations, with the latter possibly including individuals less likely to benefit from the treatment.^278^ Insufficient dosing of epacadostat, leading to suboptimal IDO1 inhibition, and the existence of alternative Trp metabolic pathways that may develop resistance to IDO1 inhibitors are also considered as factors influencing the trial outcomes.^279^, ^280^ Additionally, the dual effects of epacadostat, which may enhance IDO1's signal transduction function and induce an immunosuppressive phenotype in pDCs, could contribute to the mixed results observed in clinical trials.^281^

#### 5.2.2 Linrodostat (BMS‐986205): a potent IDO1 inhibitor

Linrodostat (BMS‐986205), an oral IDO1 inhibitor, plays a pivotal role in the early stages of the IDO1 pathway by diminishing the production of Kyn from tryptophan,^282^ showcasing greater efficacy and superior pharmacokinetic properties compared to epacadostat.^277^ Linrodostat exerts irreversible and selective inhibition on the enzymatic activity of IDO1.^283^ This agent provides irreversible and selective inhibition of IDO1's enzymatic activity. In a clinical study combining linrodostat with nivolumab for treating metastatic urothelial carcinoma, a 37% ORR and a 56% disease control rate were observed. These promising results have led to the initiation of a Phase III clinical trial for muscle‐invasive bladder cancer (NCT03661320).^282^ Linrodostat stands out as the only IDO inhibitor currently in Phase III trials, focusing on its therapeutic potential for urinary bladder neoplasms.^284^

#### 5.2.3 IDO inhibitors: expanding the therapeutic horizon

The Phase III clinical trial setback of epacadostat has undeniably shaken the confidence of many companies in the development of IDO inhibitors.^285^ However, the quest for novel IDO inhibitors continues unabated. A new generation of IDO inhibitors has been developed, encompassing dual‐target inhibitors, pan‐inhibitors targeting both IDO1 and IDO2, as well as TDO, inhibitors binding to the heme‐free form of IDO1, and proteolysis targeting chimeras of IDO1. These also include derivatives obtained by side‐chain modification of epacadostat.^280^, ^286^, ^287^ Dual‐target inhibitors, such as IDO/TDO inhibitors and IDO inhibitors targeting other pathways, have shown potential to enhance tumor treatment efficacy by achieving synergistic effects between two targets, avoiding drug interactions and metabolic disparities.^288^, ^289^, ^290^, ^291^

### 5.3 Targeting GAPDH

4‐Octyl itaconate, a cell‐permeable itaconate derivative, reduces GAPDH activity by directly alkylating cysteine residues at position 22 on the enzyme.^292^ Heptelidic acid (koningic acid) binds to the cysteine‐149 residue at the active site of GAPDH enzyme in a dosage‐dependent manner, leading to irreversible inhibition of its activity.^293^ TCH‐346 is a pharmacological agent with antiapoptotic properties that targets GAPDH, inhibiting cell apoptosis by preventing GAPDH nitrosylation and nuclear accumulation. It is primarily used for managing Parkinson's disease and motor neuron disease.^294^, ^295^, ^296^

### 5.4 Targeting ACOD1

Citraconate, an endogenous competitive inhibitor of ACOD1 and a natural isomer of itaconate, serves as a substrate analogue due to its structural similarity to cis‐aconitate. This allows citraconate to specifically bind to the active site of ACOD1, significantly inhibiting the production and accumulation of itaconate.^297^ Higher concentrations of citraconate have been detected in various disease states, including colorectal cancer and type II diabetes, highlighting its potential for inhibiting ACOD1‐mediated antitumor activity and its demonstrated anti‐inflammatory, antioxidant, and antiviral properties.^297^, ^298^

### 5.5 Targeting arginase

2‐(S)‐amino‐6‐boronohexanoic acid (ABH) marks a new era as the first arginase inhibitor to incorporate boron. Its analog, S‐(2‐boronoethyl)‐l‐cysteine (BEC), is crafted by substituting sulfur for carbon in ABH's main chain. Both ABH and BEC interact with arginase through a mechanism similar to the hydrolysis of l‐arginine.^299^ Nω‐hydroxy‐nor‐arginine (nor‐NOHA) emerges from the endogenous compound N(ω)‐hydroxy‐l‐arginine (NOHA) as an arginase inhibitor. Despite NOHA's potent arginase inhibition, its role as a transient intermediate in NOS‐mediated NO production restricts its widespread application.^300^ As nonboron analogues of ABH, both NOHA and nor‐NOHA have been shown to effectively neutralize the immunosuppressive effects of MDSCs by inhibiting arginase1, thus alleviating MDSC‐mediated T cell proliferation.^301^, ^302^ In the context of Candida infection, nor‐NOHA has demonstrated a multifaceted approach to enhancing immune responses by inhibiting arginase, regulating eosinophil infiltration, and promoting a protective type 1 immune response. This leads to an upregulation of IFN‐γ, IL‐6, TNF‐α, and other cytokines and chemokines, ultimately improving infection control and survival rates.^303^ In a model of cutaneous squamous cell carcinomas, nor‐NOHA has been shown to modulate tumor progression by enhancing T cell and DC infiltration and PD‐1 expression, with a synergistic effect observed when combined with the anti‐PD‐1 monoclonal antibody nivolumab.^303^ Currently, nor‐NOHA is being explored in clinical trials for coronary artery disease and diabetes management. The development of N‐substituted 3‐amino‐4‐(3‐boronopropyl) pyrrolidine‐3‐carboxylic acids, a novel ABH derivative with enhanced inhibitory activity against both arginase1 and arginase2, promises a more potent therapeutic option,^304^ although further research is warranted.

### 5.6 Targeting HDAC6

Despite discovering several HDAC6 inhibitors, including Nexturastat A (NextA),^305^ Tubacin,^306^ and TSA,^307^ their clinical application has been limited due to insufficient anticancer efficacy and the requirement for high drug concentrations. The development of dual‐target HDAC6 inhibitors for cancer therapy is an area of active research, with promising candidates such as dual HDAC6/PI3K inhibitors, dual HDAC6/mTOR inhibitors, dual HDAC6/BRD4 inhibitors, and dual HDAC6/HSP90 inhibitors in various stages of development. While these inhibitors are still largely experimental, they hold great promise for the future of targeted cancer therapies.^161^

## 6 DISCUSSIONS

In this comprehensive review, we have delved into the critical role of metabolic enzymes as immune checkpoints, highlighting their dual capacity to maintain physiological homeostasis or contribute to disease progression. The interplay between immunity and metabolism is undeniable, with metabolic enzymes influencing the activation and proliferation of immune cells through a delicate balance of nonenzymatic signaling and enzymatic reactions. This regulation is achieved through a sophisticated interplay between nonenzymatic signaling functions and enzymatic activities, harnessing vital nutrients and yielding regulatory metabolites. Together, these processes sculpt the contours of immune tolerance, modulating the immune response in a manner that is both precise and dynamic.

In the realm of tumor immunotherapy, two formidable challenges have emerged: the metabolic reprogramming of the tumor microenvironment and the inherent limited immunogenicity of tumors.^308^ Malignant cells often subvert the immune system's defenses by modulating the metabolic enzymes and metabolites that govern immune responses. Innovative strategies, such as introducing recombinant human arginase I (rhArg I) to induce arginine deprivation in arginine‐depleted tumors, represent a broad‐spectrum approach to cancer therapy.^147^ This approach targets the metabolic vulnerabilities of malignant cells, leveraging the metabolic milieu to enhance the efficacy of cancer treatments.

Despite the substantial advancements in immune metabolic enzyme inhibitors and complementary treatments, several areas demand further exploration. The precise mechanisms by which immune metabolic enzymes regulate immune responses require deeper investigation. For instance, the immunosuppressive effects of IDO‐induced tryptophan depletion are a subject of debate. While some studies suggest that tryptophan depletion by IDO impairs T cell function, others find no such effect in vitro. Recent evidence indicates that IDO1‐mediated tryptophan depletion may enhance tumor cell recognition by diversifying peptide fragment structures, challenging the notion that tryptophan depletion is the primary cause of immunosuppression.^309^, ^310^

Moreover, the ability of immune metabolic enzymes such as IL4I1,^113^ CD38, and IDO to upregulate PD‐1 expression raises questions about the efficacy of combined therapies targeting these enzymes with PD‐1 inhibitors. For instance, nor‐NOHA effectively induced apoptosis in arginase 2‐expressing cells under hypoxic conditions, but exhibited different effects under normoxic conditions.^311^ The interplay between CTLA‐4 and IDO, where IDO inhibition can modulate the availability of tryptophan for other pathways, further highlights the intricate molecular dynamics at play.^312^, ^313^

Ongoing research on immune metabolic enzymes and their cellular expression holds the potential to reveal innovative therapeutic approaches and theoretical insights. These discoveries could significantly bolster our therapeutic capabilities against a myriad of diseases and improve prognosis prediction. The convergence of immune metabolic checkpoint inhibitors with other immune checkpoint modulators, alongside radiotherapy, chemotherapy, and a spectrum of biological therapies, may signify a new era in cancer treatment. Such an integrated approach carries the potential to transform our combat against this multifaceted adversary.

## AUTHOR CONTRIBUTIONS

R. X. and X. H. wrote the manuscript. J. X. helped sort out part of the data. G. Y. and Y. W. designed the review and provided supervision. All authors have read and agreed to the published version of the manuscript.

## CONFLICT OF INTEREST STATEMENT

The authors declare no conflict of interest.

## ETHICS STATEMENT

Not applicable.

## Data Availability

No data were used for the research described in the article.
